# Supplementary material for: Engineered human myogenic cells in hydrogels generate innervated vascularized myofibers within dystrophic mouse muscle on long-term engraftment
Source: Cell Rep Med. 2025 Mar 7;6(3):102019. doi: 10.1016/j.xcrm.2025.102019 (PMC11970389; doi:10.1016/j.xcrm.2025.102019)
Supplement: Document S1. Tables S1–S5 [file mmc1.pdf]

Cell Reports Medicine, Volume *6*

Supplemental information

Engineered human myogenic cells in hydrogels generate innervated vascularized myofibers within dystrophic mouse muscle on long-term engraftment

Anna Kowala, James Boot, Jinhong Meng, Charles A. Mein, Olivier Pourquié, John T. Connelly, Jennifer E. Morgan, and Yung-Yao Lin

Table S1. Quantification of transplantation experiments at 4 weeks (Related to Figure 3 and 4).

Experiment no.	Timepoint of TA collection	Condition	Number of cells/myofibers detected by human-specific antibodies						
			Undifferentiated cells		Differentiated human myofibers				
			hLaminA/C+		hDys+	hSpectrin+, hDys+, hLaminA/C+	hSpectrin+, hDys+	hSpectrin+, hLaminA/C+	Total
1	4 weeks	CORR-R3381X MPCs in Promocell	2		0	2	2	0	4
			4		0	5	0	0	5
			1		0	2	0	0	2
			1		0	0	1	0	1
			10		1	16	10	0	27
			1		0	2	0	0	2
		hCD133+ in Megacell	6		1	26	17	0	44
			18		9	45	49	0	103
			9		0	1	1	0	2
			36		47	156	74	0	277
			4		11	22	20	0	53
			16		9	85	28	0	122
2	4 weeks	CORR-R3381X MPCs in Promocell	4		2	5	13	0	20
			13		13	64	100	0	177
			1		0	3	3	0	6
			5		4	6	25	0	35
			0		0	5	8	0	13
		CORR-R3381X MPCs in Megacell	0		0	0	0	0	0
			1		3	0	2	0	5
			1		0	0	2	0	2
			2		1	2	4	0	7
			0		2	2	3	0	7
			0		1	2	4	0	7
3	4 weeks	CORR-R3381X MPCs in Promocell	7		1	5	13	1	20
			10		1	16	22	0	39
			12		2	29	66	2	99
			4		3	23	88	1	115
		CORR-K295fs MPCs in Promocell	6		1	5	19	0	25
			0		0	20	40	0	60
			3		0	3	7	0	10
			2		0	3	10	0	13
			2		0	5	19	0	24
4	4 weeks	CORR-K295fs MPCs in Promocell	0		0	0	2	0	2
			12		0	0	3	0	3
			5		1	0	7	0	8
			3		0	0	2	0	2
		CORR-K295fs MPCs in Megacell	5		0	1	1	0	2
			1		2	3	5	0	10
			5		0	1	0	0	1
5	4 weeks	CORR-K295fs MPCs in Promocell	1		0	0	0	0	0
			4		0	0	0	0	0
			3		0	1	0	0	1

Table S2. Quantification of transplantation experiments at 5 and 6 months (Related to Figure 5).

Timepoint of TA collection	Condition	Number of cells/myofibers detected by human-specific antibodies						
		Undifferentiated cells		Differentiated human myofibers				
		hLaminA/C+		hDys+	hSpectrin+, hDys+, hLaminA/C+	hSpectrin+, hDys+	hSpectrin+, hLaminA/C+	Total
5 months	CORR-R3381X MPCs in Promocell	1		25	12	61	0	98
6 months	CORR-R3381X MPCs in Promocell	0		0	0	0	0	0
		0		14	6	8	0	28
		0		0	0	0	0	0
		0		22	15	22	0	59
		0		0	0	0	0	0

Table S3. Descriptive statistics for cross-section areas (CSAs) of donor-derived hMyofibers (Related to Figure 6).

	CORR-R3381X (4 weeks)	hCD133+ (4 weeks)	CORR-R3381X (5 & 6 months)
Total number of hMyofibers	391	518	185
CSA (μm^2)			
Minimum	26.19	21.23	29.21
25% Percentile	181.6	101	174.5
Median	317.2	172.5	344.5
75% Percentile	494.7	302.6	577.5
Maximum	2933	1600	3055
Range	2906	1579	3025
Mean	400.3	242.7	420.8
Std. Deviation	334.4	223.7	351.4
Std. Error of Mean	16.91	9.828	25.84

Table S4. Relative frequency distribution (percentage) (Related to Figure 6).

Bin Center (μm^2)	CORR-R3381X (4 weeks) (%)	hCD133+ (4 weeks) (%)	CORR-R3381X (5 & 6 months) (%)
50	15.08951407	44.59459459	17.83783784
250	40.15345269	35.52123552	32.97297297
450	26.342711	12.93436293	21.62162162
650	7.16112532	3.474903475	14.59459459
850	5.626598465	1.544401544	5.945945946
1050	2.046035806	0.772200772	4.324324324
1250	1.023017903	0.386100386	1.621621622
1450	1.023017903	0.386100386	0
1650	0.767263427	0.386100386	0.540540541
1850	0	0	0
2050	0.511508951	0	0
2250	0	0	0
2450	0	0	0
2650	0	0	0
2850	0.255754476	0	0
3050	0	0	0.540540541

Table S5. Cell culture medium and hydrogel recipes (Related to Figure 2 - 6).

#	Name	Details
1	Promocell Skeletal Muscle Growth medium (C-23060)	95 ml of basal medium (C-23060B), 5 ml of Supplement Mix (C-39365) and 105 µl 0.2% Penicillin/Streptomycin (Gibco, 15140122)
2	Megacell Skeletal Muscle Growth Medium	MegaCell Dulbecco′s Modified Eagle′s Medium (Sigma-Aldrich, M3942) supplemented with 10% fetal bovine serum (FBS, Gibco, 10270-098), 0.1mM β-Mercaptoethanol (Gibco, 31350-010), 1% NEAA (Gibco, 11140-035), 2µM Glutamine (GlutaMAX™ Supplement, Thermo Fisher, 35050061) and 5 ng/ml recombinant FGF-2 (PeproTech, 100-18B)
3	Fibrin/Matrigel hydrogel	4 mg/ml fibrinogen (40% vol; Sigma, F8630-1G), Matrigel (3.5 mg/ml; Fisher Scientific, 11543550) in DMEM/F12 media (40% vol; Invitrogen, 11320-033), thrombin (Sigma, T6634; 0.2 unit/mg fibrinogen)
