# Supplementary material for: Engineered human myogenic cells in hydrogels generate innervated vascularized myofibers within dystrophic mouse muscle on long-term engraftment
Source: Cell Rep Med. 2025 Mar 7;6(3):102019. doi: 10.1016/j.xcrm.2025.102019 (PMC11970389; doi:10.1016/j.xcrm.2025.102019)
Supplement: Document S2. Article plus supplemental information [file mmc5.pdf]

Cell Reports Medicine

Article

Engineered human myogenic cells in hydrogels generate innervated vascularized myofibers within dystrophic mouse muscle on long-term engraftment

Graphical abstract

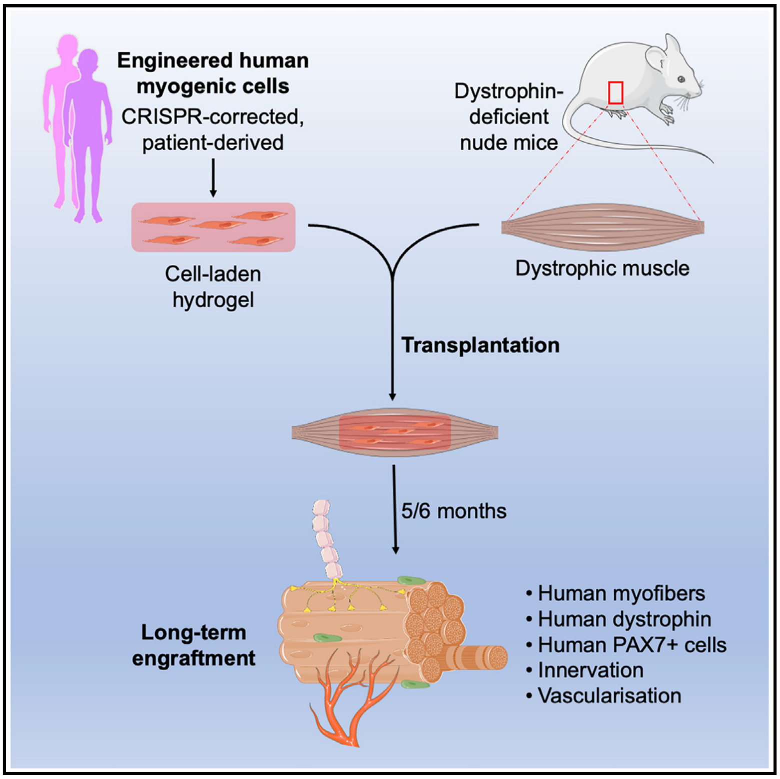

Highlights

- A clinically relevant transplantation strategy without modulation of host muscles
- Engineered human myogenic cells in hydrogels are engraftable and safe
- Long-term engraftment with innervation and vascularization in dystrophic mice
- Human PAX7+ cells populate the satellite cell niche

Authors

Anna Kowala, James Boot, Jinhong Meng, ..., John T. Connelly, Jennifer E. Morgan, Yung-Yao Lin

Correspondence

yy.lin@qmul.ac.uk

In brief

Kowala et al. demonstrate a clinically relevant transplantation strategy synergizing engineered human myogenic cells and hydrogel-mediated delivery in a dystrophic mouse model. Upon long-term engraftment, dystrophin-positive human myofibers are innervated by mouse motor neurons and supported by vascularization within dystrophic mouse muscle. Human PAX7+ cells populate muscle stem cell niche.

Kowala et al., 2025, Cell Reports Medicine 6, 102019
March 18, 2025 © 2025 The Author(s). Published by Elsevier Inc.
https://doi.org/10.1016/j.xcrm.2025.102019

CellPress

Cell Reports Medicine

CellPress
OPEN ACCESS

Article

Engineered human myogenic cells in hydrogels generate innervated vascularized myofibers within dystrophic mouse muscle on long-term engraftment

Anna Kowala,[1,2,3,4] James Boot,[5] Jinhong Meng,[4,8] Charles A. Mein,[5] Olivier Pourquié,[6] John T. Connelly,[3,7] Jennifer E. Morgan,[4,8,9] and Yung-Yao Lin[1,2,3,9,10,*]

[1]Centre for Genomics and Child Health, Blizard Institute, Faculty of Medicine and Dentistry, Queen Mary University of London, 4 Newark Street, London E1 2AT, UK
[2]Stem Cell Laboratory, National Bowel Research Centre, Blizard Institute, Faculty of Medicine and Dentistry, Queen Mary University of London, 2 Newark Street, London E1 2AT, UK
[3]Centre for Predictive in vitro Models, Queen Mary University of London, Mile End Road, London E1 4NS, UK
[4]UCL Great Ormond Street Institute of Child Health, 30 Guilford Street, London WC1N 1EH, UK
[5]Barts and the London Genome Centre, Faculty of Medicine and Dentistry, Blizard Institute, London, UK
[6]Department of Genetics, Harvard Medical School and Department of Pathology, Brigham and Women's Hospital, 60 Fenwood Road, Boston, MA, USA
[7]Centre for Cell Biology and Cutaneous Research, Blizard Institute, Faculty of Medicine and Dentistry, Queen Mary University of London, 4 Newark Street, London E1 2AT, UK
[8]NIHR Biomedical Research Centre at Great Ormond Street Hospital, Great Ormond Street, London, UK
[9]Senior author
[10]Lead contact
*Correspondence: yy.lin@qmul.ac.uk
https://doi.org/10.1016/j.xcrm.2025.102019

SUMMARY

Transplantation of human myogenic progenitor cells (MPCs) is a promising therapeutic strategy for treating muscle-wasting diseases, e.g., Duchenne muscular dystrophy (DMD). To increase engraftment efficiency of donor stem cells, modulation of host muscles is required, significantly limiting their clinical translation. Here, we develop a clinically relevant transplantation strategy synergizing hydrogel-mediated delivery and engineered human MPCs generated from CRISPR-corrected DMD patient-derived pluripotent stem cells. We demonstrate that donor-derived human myofibers produce full-length dystrophin at 4 weeks and 5–6 months (long-term) after transplantation in the unmodulated muscles of the dystrophin-deficient mouse model of DMD. Remarkably, human myofibers are innervated by mouse motor neurons forming neuromuscular junctions and supported by vascularization after long-term engraftment in dystrophic mice. PAX7+ cells of human origin populate the satellite cell niche. There was no evidence of tumorigenesis in mice engrafted with hydrogel-encapsulated human MPCs. Our results provide a proof of concept in developing hydrogel-based cell therapy for muscle-wasting diseases.

INTRODUCTION

Skeletal muscle is responsible for the generation of all voluntary movements, such as walking and lifting objects. Skeletal muscle has a remarkable regenerative capacity mediated by resident muscle satellite cells.[1–3] Muscular dystrophies are a group of genetic muscle-wasting conditions characterized by cycles of myofiber degeneration and regeneration with accumulation of fat and connective tissue. Duchenne muscular dystrophy (DMD), caused by dystrophin deficiency, is the most common form of muscular dystrophy in childhood, resulting in loss of ambulation, poor quality of life, and premature death.[4] Currently there is still no cure for any form of muscular dystrophies.

A potential approach for treating muscular dystrophies is cell-based therapy. A variety of stem/progenitor cells with myogenic potential have been used in previous studies to restore dystrophin expression in dystrophin-deficient mouse muscles, including satellite cells,[5,6] myoblasts,[7,8] pericytes,[9] and human skeletal muscle-derived CD133+ (hCD133+) cells,[10,11] as well as mesoangioblasts in dystrophin-deficient dogs.[12] However, one of the major challenges in cell-based therapies for muscular dystrophies is obtaining sufficient number of stem/progenitor cells with myogenic potential for effective engraftment. This hurdle can now be addressed using human pluripotent stem cells (PSCs), embryonic stem cells (ESCs) or induced pluripotent stem cells (iPSCs), with transgene dependent or transgene-free differentiation protocols to generate an unlimited supply of myogenic progenitor cells (MPCs) for transplantation.[13,14] Using a transcription factor PAX7-based fluorescence reporter, studies have shown that human PSC-derived MPCs are engraftable as they can not only

© 2025 The Author(s). Published by Elsevier Inc.
This is an open access article under the CC BY license (http://creativecommons.org/licenses/by/4.0/).

give rise to dystrophin-positive myofibers but also reconstitute the satellite cell compartment within the host mouse muscles.[15–17]

Conventionally, donor cells with myogenic potential were transplanted into immunodeficient host animals through intramuscular cell injection. To increase engraftment efficiency, the host muscles were modulated prior to direct injection of donor cells using a range of regimens, including irradiation, cryoinjury, barium chloride ($BaCl_2$), and myotoxins such as notexin and cardiotoxin. It was shown that deprivation of endogenous satellite cells in combination with preservation of host satellite cell niche significantly augmented donor mouse satellite cell engraftment.[18] Apart from modulation of host muscles, it was shown that engraftment efficiency could also be affected by donor cell types and the recipient host animal strains that have different levels of immunodeficiency.[19] Thus, both intrinsic properties of donor cells and local environment of host tissues are important factors in determining engraftment efficiency. Moreover, the microenvironment of dystrophic muscle may inhibit donor cell survival and differentiation as shown in the clinical trials.[20,21] Despite progress demonstrated in previous proof-of-principle studies, delivery of donor cells via intramuscular injection is considered invasive and not practical in clinical settings as hundreds of injections would be required to cover large regions of skeletal muscle.[22,23] In this regard, transplantation of donor cells via intramuscular injection significantly limits their clinical translation. Therefore, it is important to develop better transplantation methods to deliver human myogenic cells into host muscles for improving engraftment efficiency.

Tissue engineering approaches have been utilized to generate natural or synthetic biomaterial 3D scaffolds providing mechanical characteristics and signaling cues required for proliferation and differentiation of muscle stem/progenitor cells.[24–26] To date, few studies have reported transplantation of human myogenic cell-laden hydrogels into host animals, including non-obese diabetic (NOD) severe combined immunodeficiency (SCID) gamma (NSG) mice,[27–29] SCID mice,[30] nude mice,[31] and Rowett nude (RNU) rats.[31,32] In general, these studies reported the short-term (1–8 weeks) engraftment potential of the 3D scaffold systems with some promising signs of innervation and vascularization in the engrafted regions. However, it remains an open question whether the biomaterial 3D scaffolds can support engraftment of human myogenic cells in dystrophin-deficient dystrophic animal models by demonstrating that donor-derived human myofibers produce dystrophin. Furthermore, even though Rao et al. observed PAX7+ cells adjacent to myotubes within the implants at 2–3 weeks after transplantation,[28] it remains to be demonstrated whether these PAX7+ cells were of donor or host origin or both and whether PAX7+ cells of human origin could enter the satellite cell compartment in host animals. Finally, the long-term (up to 6 months) engraftment potential of human myogenic cell-laden hydrogels and their safety regarding tumorigenesis should also be investigated.

We previously generated two independent sources of human MPCs from two precisely CRISPR-corrected DMD patient-derived PSC lines (CORR-R3381X and CORR-K2957fs) and demonstrated the restoration of full-length dystrophin (Dp427) *in vitro*.[33,34] In this study, we assess the *in vivo* regenerative potential of human CORR-R3381X or CORR-K2957fs MPCs compared with skeletal muscle-derived hCD133+ cells[11] by encapsulating them in fibrin/Matrigel hydrogels, followed by transplantation into dystrophin-deficient *mdx* nude mice. We show that engineered human myogenic cells contribute to muscle regeneration *in vivo* and express full-length dystrophin as demonstrated by quantification of human-specific antibodies against key markers. Importantly, we demonstrate innervation and vascularization of human myofibers in the engrafted regions, PAX7+ cells of human origin populating the satellite cell niche, and no evidence for tumorigenesis after long-term xenoengraftment (5–6 months). Together, our study provides a clinically relevant strategy synergizing engineered human PSC-derived MPCs and hydrogel-mediated delivery for developing cell therapies to treat muscle-wasting conditions, such as DMD.

RESULTS

Transcriptome analysis of DMD and CRISPR-corrected MPCs identifies differentially enriched signaling pathways required for muscle stem cell function

We previously performed two independent myogenic transcriptome analyses using two isogenic pairs of human PSC-derived DMD and CRISPR-corrected myogenic cultures, i.e., DMD-R3381X and CORR-R3381X,[33] as well as DMD-K2957fs and CORR-K2957fs.[34] While both DMD mutations disrupt the full-length dystrophin protein (Dp427 isoform), the K2957fs mutation spares the Dp71 isoform. Broadly speaking, our *in vitro* studies showed reduced myogenic differentiation competence in DMD MPCs compared to CORR MPCs. Profiling the molecular signatures of DMD and CORR MPCs enables a systems biology approach to identify differentially enriched signaling pathways and biological processes. Thus, we decided to re-analyze transcriptomes of the undifferentiated MPCs (day 0, cultured in growth medium) using both datasets (GSE159273 and GSE189053).

We first took the raw RNA sequencing (RNA-seq) reads from day 0 samples from each dataset and re-aligned the reads to reference human genome (version GRCh38.104), using the Spliced Transcripts Alignment to a Reference (STAR) aligner.[35] Between DMD and CORR MPC transcriptomes (day 0), we identified 1,626 differentially expressed (DE) genes (Data S1). The heatmap of these DE genes showed clustering of the CORR group (CORR-R3381X and CORR-K2957fs) and the DMD group (DMD-R3381X and DMD-K2957fs), and the sub-clustering within each group was determined by the original MPC genotypes, reflecting the interindividual variability (Figure 1A). Next, we performed gene-list functional profiling using gProfiler[36] and identified statistically significantly enriched gene ontology (GO) terms (Data S2). Consistent with our previous studies,[33,34] the top 5 GO terms are related to muscle cell differentiation and development (Figure 1B). Interestingly, the gProfiler analysis also revealed that signaling pathways attributed to skeletal muscle stem cell function[37] were differentially enriched in DMD MPCs, such as p38 mitogen-activated protein kinase (MAPK) signaling,[38,39] Jun N-terminal kinase (JNK) signaling,[40] Wnt signaling,[41] notch signaling,[42] and regulation of mitotic spindle/cell cycle.[43] In agreement with the gProfiler analysis, gene set enrichment analysis (GSEA)[44] showed that gene sets involved in muscle stem

CellPress
OPEN ACCESS

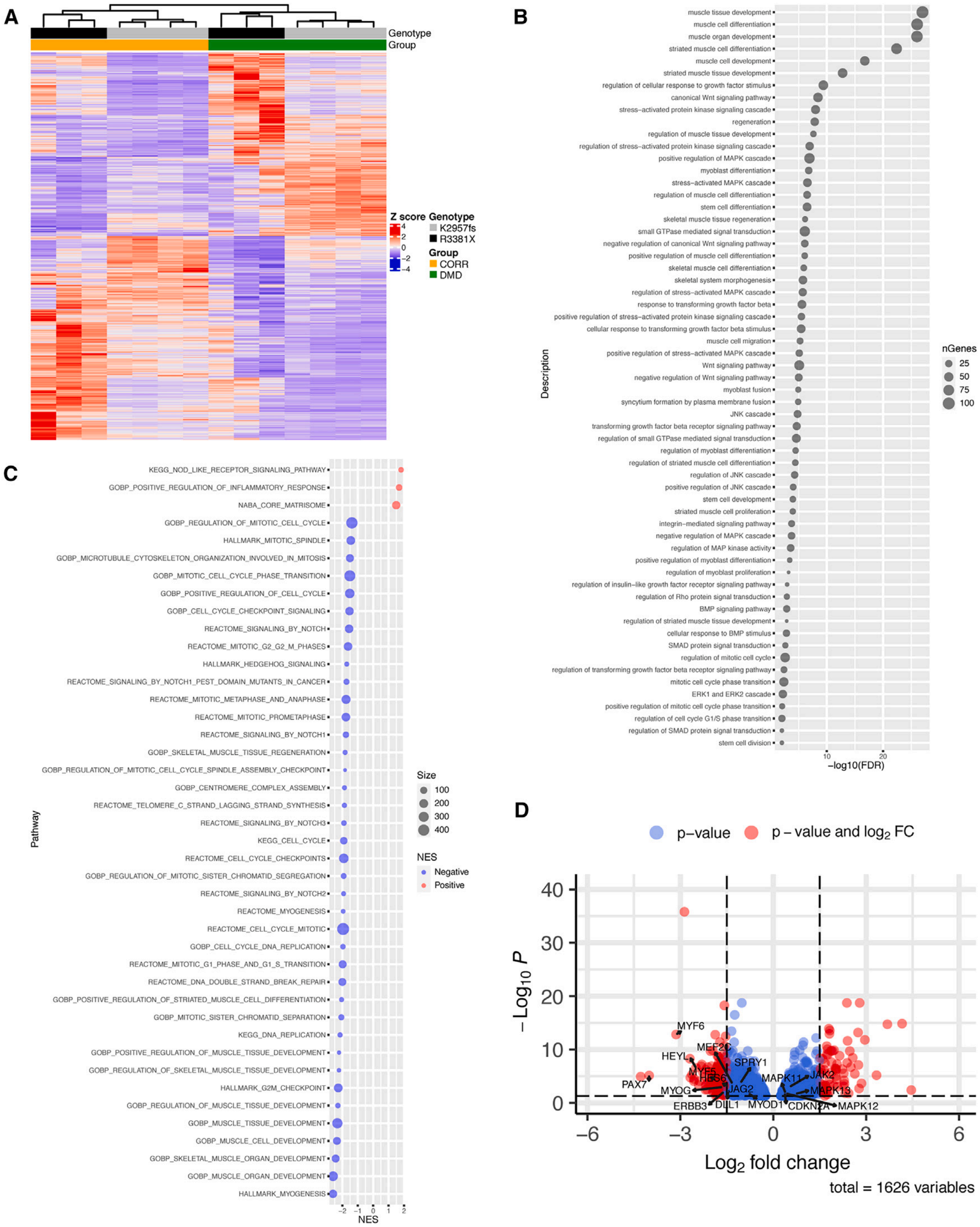

Figure 1. Signaling pathways required for muscle stem cell function are differentially enriched in DMD MPCs compared to CRISPR-corrected MPCs

(A) Heatmap of all DE genes between undifferentiated DMD and CORR MPCs (day 0, cultured in growth medium). FDR < 0.05. 3 biological replicates for the DMD-R3381X and CORR-R3381X isogenic pair and 4 biological replicates for the DMD-K2957fs and CORR-K2957fs isogenic pair. See also Data S1.

(B) Bubble plot of selected signaling pathways from gProfiler analysis. Bubbles sized by pathway size. See also Data S2.

(C) Bubble plot of normalized enrichment scores (NESs) of selected gene sets negatively and positively enriched in DMD versus CORR MPCs from GSEA (adjusted $p < 0.05$). Bubbles sized by pathway size. See also Data S3.

(D) Volcano plot shows DE genes (DMD versus CORR) with genes of interest annotated. DE genes with an FDR < 0.05 and a log fold change <−1.5 or >1.5 are labeled red, while genes only meeting the FDR threshold are labeled blue.

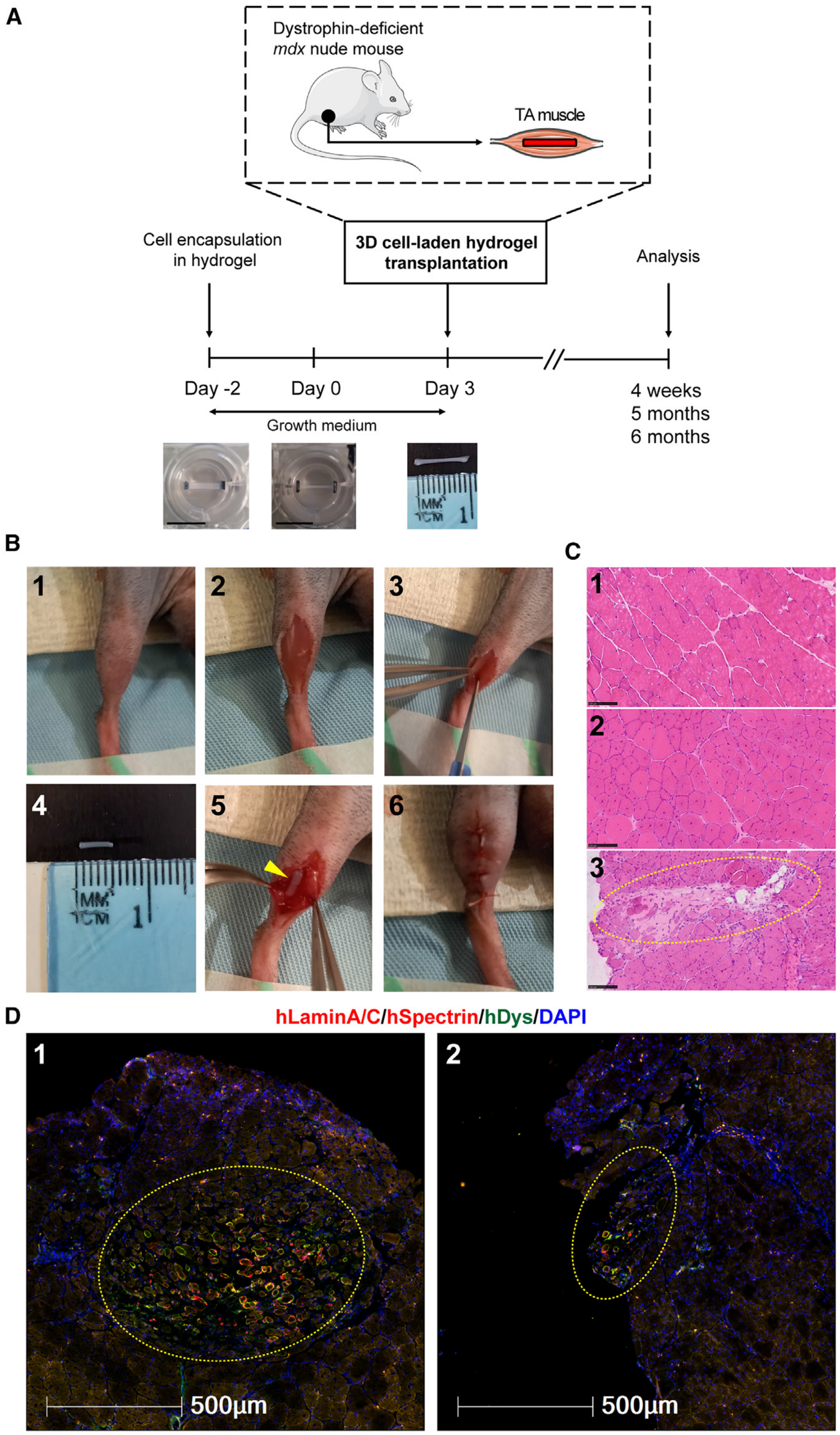

Figure 2. Experimental timeline and xenoengraftment of engineered human 3D cell-laden constructs in *mdx* nude mice

(A) Following encapsulation of human PSC-derived MPCs in hydrogel in PDMS molds, 3D cell-laden constructs were cultured for 5 days in growth medium (either Promocell or Megacell) and then transplanted into TA muscles of *mdx* nude mice, followed by analysis at 4 weeks, 5 months, or 6 months after xenoengraftment. Scale bars, 10 mm.

(B) Step-by-step transplantation procedure: (1) positioning of the hindlimb; (2) skin incision to reveal TA muscle; (3) TA muscle incision with a scalpel; (4) prior to transplantation, hydrogel with encapsulated cells was cut with 5 mm-diameter biopsy punch and removed from its PDMS mold; (5) placement of a 3D construct into TA muscle; and (6) closure of the muscle incision, with a 3D construct inside, and suture of the skin.

(C) Representative H&E staining of transverse cryosections of TA muscles. Hematoxylin (purplish blue) stains cell nuclei, and eosin (pink) stains the extracellular matrix and cytoplasm. Panel 1, C57Bl/10 mouse (non-dystrophic control) with nuclei located at the periphery of myofibers; panel 2, non-transplanted *mdx* nude (dystrophic control). Central nuclei within the myofibers are characteristic of *mdx* nude mouse muscles. Panel 3, at 4 weeks after transplantation, the transplanted hydrogel construct (dashed yellow circle) is visible within *mdx* nude TA muscle. Scale bars, 100 μm.

(D) Representative images of hydrogel-based engraftment of human myogenic cells in TA muscle of *mdx* nude mice at 4 weeks after transplantation. Engrafted cells and myofibers of human origin in the middle of mouse TA muscle (dashed yellow circles, panel 1) or at the edge of mouse TA muscle (dashed yellow circles, panel 2) are shown. Transverse 10 μm sections were stained with antibodies against human lamin A/C and human spectrin (both red) and human dystrophin (green). Nuclei were counterstained with DAPI (blue). Scale bars, 500 μm.

cell function that are negatively enriched in DMD MPCs include MYOGENESIS, NOTCH signaling, G2M CHECKPOINT, and MITOTIC SPINDLE (Data S3; Figure 1C). In contrast, POSITIVE REGULATION OF INFLAMMATORY RESPONSE, NOD LIKE RECEPTOR SIGNALING PATHWAY, and CORE MATRISOME (extracellular matrix proteins and regulators) are positively enriched in DMD MPCs (Data S3; Figure 1C).

Consistent with the immunocytochemistry results in our previous studies,[33,34] the volcano plot showed that the transcription factor *PAX7* transcripts were significantly down-regulated in DMD MPCs compared to CORR MPCs (Figure 1D). In addition, genes involved in myogenesis (*MYF5*, *MYOD1*, *MYOG*, *MYF6*, *MEF2C*, and *ERBB3*),[45,46] regulation of muscle stem cell self-renewal (*SPRY1*),[37,47] and NOTCH signaling (*DLL1*, *JAG2*, *HES6*, and *HEYL*)[37] were also significantly down-regulated, whereas genes involved in p38 MAPK signaling (*MAPK11* [p38β] and *MAPK13* [p38δ]),[48] regulation of muscle

CellPress
OPEN ACCESS

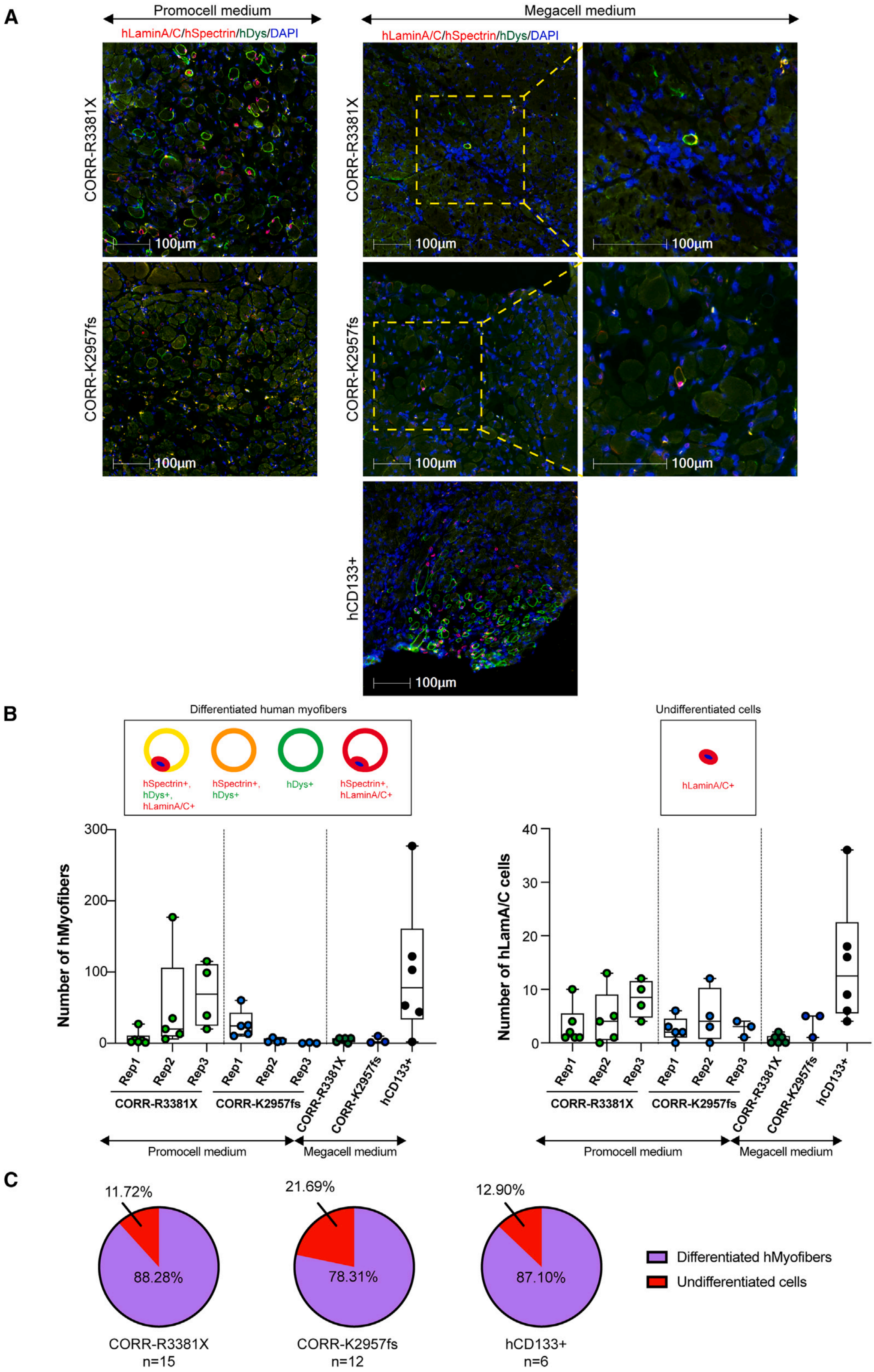

(legend on next page)

stem cell commitment (*MAPK12* [p38γ]),[49] inflammatory response (*JAK2*),[50] and senescence (*CDKN2A* [p16^{INK4a}])[51] were significantly up-regulated in DMD MPCs compared to CORR MPCs (Figure 1D).

Taken together, our results suggest that signaling pathways involved in muscle stem cell function are dysregulated in DMD MPCs, which may contribute to the eventual failure of skeletal muscle regeneration in DMD patients. Since both CORR MPCs have the DMD mutations precisely corrected, we hypothesize that they have good regenerative potential *in vivo*. We then sought to test whether the CORR MPCs are engraftable in dystrophin-deficient *mdx* nude mice.

Successful xenoengraftment by transplanting human cell-laden hydrogel constructs into dystrophin-deficient mice

To develop a clinically relevant transplantation protocol, we decided not to modulate host mouse muscles using irradiation, cryoinjury, $BaCl_2$, or any myotoxin prior to donor human cell transplantation. In addition, we employed an established method to generate engineered 3D cell-laden constructs in polydimethylsiloxane (PDMS) molds[24] and investigated the regenerative potential of CRISPR-corrected human PSC-derived MPCs *in vivo*. Briefly, we encapsulated human myogenic cells in fibrin/Matrigel-based hydrogel cultured in growth medium (day −2). The 3D cell-laden constructs remodeled within 2 days (day 0). We then transplanted these 3D cell-laden constructs into dystrophin-deficient *mdx* nude mice (day 3), followed by analysis at specific time points (Figure 2A). By placing hindlimbs in a position allowing access to the tibialis anterior (TA) muscles, we performed longitudinal skin and TA muscle incisions. In the meantime, the engineered 3D cell-laden constructs were cut with a 5 mm biopsy punch and removed from PDMS molds for transplantation. Next, a 3D cell-laden construct was embedded within the incision of the TA muscles, and the skin incision was sutured (Figure 2B).

As a control, we used healthy human skeletal muscle-derived hCD133+ cells (a subset of satellite cells), previously demonstrated as capable of contributing to muscle regeneration and forming functional satellite cells after intramuscular transplantation in both immunodeficient Rag2-/γ chain-/C5- and *mdx* nude mice.[11,19] For comparison with hCD133+ cells, we used two independent CRISPR-corrected human PSC-derived MPCs (CORR-R3381X and CORR-K2957fs). Prior to transplantation, cell-laden 3D constructs were cultured in different growth medium conditions for 5 days. At 4 weeks after transplantation, hematoxylin and eosin (H&E) staining showed the presence of hydrogel implants within TA muscle of *mdx* nude mice (Figure 2C). Among 56 analyzed TA muscles, immunostaining of muscle sections with human-specific antibodies (hLaminA/C, hSpectrin, and hDystrophin) revealed myofibers of human origin either in the middle (64.29%) or at the edge (32.14%) of the host TA muscle (Figure 2D). Only 3.57% of the analyzed TA muscles contained no myofibers of human origin. Importantly, transverse sections of host muscles contained not only hLaminA/C+ nuclei (of human origin) but also hSpectrin+ and hDystrophin+ myofibers (Figure 2D), demonstrating *in vivo* skeletal muscle regeneration from engineered human myogenic cells. Together, these results indicate successful xenoengraftment of human myogenic cells in dystrophin-deficient *mdx* nude mice by hydrogel-mediated delivery.

CORR-R3381X and CORR-K2957fs MPCs contribute to muscle regeneration *in vivo* similar to skeletal muscle-derived hCD133+ cells

Next, we sought to compare the efficiency of *in vivo* muscle regeneration between different experimental conditions by quantifying myofibers of human origin. Notably, it was shown that newly regenerated myofibers in *mdx* nude, *NOD/Rag1(null) mdx(5cv)*, or *NOD/LtSz-SCID IL2Rγ(null)* mice were recognized by anti-human spectrin antibody, which might be due to expression of utrophin in regenerating myofibers.[52] This might lead to the detection of false-positive donor myofibers. Moreover, after transplantation into host muscles, myogenic cells might not only fuse with each other forming myofibers of donor origin but also fuse with host myogenic cells or host myofibers, resulting in mosaic myofibers.[53] Therefore, spectrin and dystrophin immunostaining might show a mosaic pattern.[53,54] To interpret data cautiously, we decided to quantify myofibers of human origin as hSpectrin+ myofibers containing hLaminA/C+ nuclei and any hDystrophin+ myofibers. In addition, hLaminA/C+ nuclei that were not within a myofiber containing hSpectrin or hDystrophin might have been either satellite cells or undifferentiated cells of human origin.

In total, we compared 5 experimental conditions, in which CORR-R3381X and CORR-K2957fs cell-laden constructs were cultured in either Promocell or Megacell growth medium for 5 days after encapsulation, whereas hCD133+ cell-laden constructs were cultured in Megacell growth medium. In agreement with previous studies in which cells were transplanted into modulated host muscles,[11,19] we showed that all hCD133+ cell-laden constructs contributed to muscle regeneration *in vivo* at 4 weeks after transplantation (Figure 3A; Table S1).

Figure 3. Engrafted cells and myofibers of human origin in *mdx* nude mice at 4 weeks after transplantation

(A) Representative transverse cryosections of *mdx* nude mouse TA muscle transplanted with 3D constructs of CORR-R3381X MPCs in Promocell growth medium, CORR-K2957fs MPCs in Promocell growth medium, CORR-R3381X MPCs in Megacell growth medium, CORR-K2957fs MPCs in Megacell growth medium, and hCD133+ cells in Megacell growth medium. At 4 weeks after transplantation, transverse 10 μm sections were stained with antibodies against human lamin A/C and human spectrin (both red) and human dystrophin (green). Nuclei were counterstained with DAPI (blue). Scale bars, 100 μm.

(B) Quantification of myofibers and undifferentiated cells of human origin in each experimental condition or independent replicate (Rep 1, 2, and 3). Schematics represent immunostaining patterns considered as myofibers of human origin (hMyofibers) or undifferentiated cells of human origin (hLamin A/C+ only). Values indicate minimum, maximum, median, and 25th and 75th percentiles.

(C) Pie charts show percentages of engrafted human myogenic cells in *mdx* nude mice as undifferentiated cells and differentiated human myofibers. CORR-R3381X MPCs in Promocell growth medium (15 TA muscles), CORR-K2957fs MPCs in Promocell growth medium (12 TA muscles), and hCD133+ cells in Megacell growth medium (6 TA muscles). In Promocell medium: CORR-R3381X, 15 biological replicates. CORR-K2957fs, 12 biological replicates. In Megacell medium: CORR-R3381X, 6 biological replicates. CORR-K2957fs, 3 biological replicates; hCD133+, 6 biological replicates.

Nonetheless, it should be noted that the number of human myofibers varies widely in the host mice, ranging from 2 to 277 human myofibers in host TA muscles (Figure 3B; Table S1), which was consistent with the transplantation efficiency of the same type of cells (without hydrogel encapsulation) into irradiated and cryoinjured *mdx* nude mouse muscle.[11,19] In contrast, CORR-R3381X and CORR-K2957fs cell-laden constructs cultured in Megacell medium generated up to 7 and 10 human myofibers, respectively, in one host TA muscle (Figure 3B; Table S1). However, we found that both CORR-R3381X and CORR-K2957fs cell-laden constructs cultured in Promocell medium gave rise to higher number of donor-derived human myofibers than those cultured in Megacell medium (Figure 3B). Among three independent sets of transplantations, CORR-R3381X constructs generated up to 177 human myofibers in one host TA muscle, whereas CORR-K2957fs constructs could produce up to 60 human myofibers in one host TA muscle (Table S1). Among the independent experiments, the greatest number of myofibers of human origin per host TA muscle (median [25th–75th percentile]) from hCD133+, CORR-R3381X, and CORR-K2957fs cell-laden constructs were 78 [33.5–160.8], 69 [24.75–111], and 24 [11.5–42.5], respectively (Figure 3B; Table S1). Next, we pooled three sets of data together to investigate the percentages of undifferentiated cells (hLaminA/C+ only) and myofibers of human origin generated by CORR-R3381X or CORR-K2957fs constructs in Promocell medium and hCD133+ constructs in Megacell medium. In general, CORR-R3381X and hCD133+ constructs showed very similar percentages of undifferentiated cells (~11.72%–12.9%) and human myofibers (~87.1%–88.28%), whereas CORR-K2957fs constructs showed a higher percentage of undifferentiated cells (~21.69%) and lower percentage of human myofibers (~78.31%) (Figure 3C).

Taken together, even though the engraftment efficiencies vary widely between cell-laden constructs within the same experiment, as well as between constructs in different sets of experiments, our results demonstrated that CORR-R3381X and CORR-K2957fs MPCs can contribute to muscle regeneration *in vivo*, like skeletal muscle-derived hCD133+ cells. The fact that cell-laden constructs maintained in Promocell medium gave markedly higher engraftment efficiency than those maintained in Megacell medium suggests that engraftment efficiency could be further improved by modulating human myogenic cells with appropriate growth medium prior to transplantation.

CORR-R3381X and CORR-K2957fs MPCs gave rise to similar distribution of mosaic patterns of donor-derived human myofibers as hCD133+ cells

We subsequently investigated whether the number of donor-derived human myofibers is similar between CORR-R3381X, CORR-K2957fs, and hCD133+ cell-laden constructs. To do this, we quantified the number of donor-derived human myofibers in 4 categories, including (1) hDystrophin+ only, (2) hSpectrin+ with hDystrophin+ and hLaminA/C+, (3) hSpectrin+ with hDystrophin+, and (4) hSpectrin+ with hLaminA/C+ (Figure 4A; Table S1). Regardless of the myogenic cell sources and engraftment efficiencies, we found that >99.29% donor-derived human myofibers in representative transverse cryosections of dystrophin-deficient *mdx* nude mouse TA muscles express human dystrophin (Figure 4B). Moreover, CORR-R3381X and CORR-K2957fs constructs gave rise to very similar percentage distribution of human-specific markers in myofibers in post-transplantation TA muscle sections with the donor-derived human myofibers expressing hSpectrin+ with hDystrophin+ (~62.12%–73.65%), hSpectrin+ with hDystrophin+ and hLaminA/C+ (~25%–32.39%), or hDystrophin+ only (~1.35%–4.78%) (Figure 4B). These results suggest that CRISPR-corrected human PSC-derived MPCs may contribute to muscle regeneration after xenotransplantation in a similar way to other muscle precursor cells of human origin, such as hCD133+ cells.

Long-term engraftment of human myofibers with human PAX7+ cells populating the satellite cell niche supported by innervation and vascularization in *mdx* nude mice

Next, we sought to test whether 3D cell-laden constructs might support long-term engraftment of human myogenic cells and whether there is any concern of tumorigenesis. Given that CORR-R3381X cell-laden constructs gave better engraftment efficiency than CORR-K2957fs constructs at 4 weeks after transplantation, we decided to focus on transplantation of CORR-R3381X constructs cultured in Promocell medium, followed by analysis at either 5 ($n = 1$) or 6 ($n = 5$) months. In total, we transplanted 6 cell-laden constructs into 6 TA muscles of *mdx* nude mice. Encouragingly, donor-derived human myofibers were detected in one TA muscle that was analyzed at 5 months after transplantation (98 human myofibers) and two TA muscles at 6 months after transplantation (up to 59 human myofibers), as evidenced by hDystrophin+ staining (Figure 5; Table S2). Importantly, there is no evidence for tumorigenesis in any mouse transplanted with hydrogel-encapsulated human MPCs after long-term engraftment.

By examining transverse cryosections stained with antibodies against hLaminin B1, hSpectrin, and hLamin A/C, as well as the canonical satellite cell marker PAX7, we identified hLamin A/C and Pax7 double-positive cells of human origin residing in the satellite cell niche (underneath the basal lamina of myofibers) in the TA host muscle (Figure 5B, panel a). In addition, we could also identify cells that were hLamin A/C negative and Pax7 positive close to the engrafted region in mouse TA muscle, indicating that these were satellite cells of mouse origin (Figure 5B, panel b). Together, these results suggest that transplantation of 3D cell-laden hydrogel constructs not only contributes to muscle regeneration *in vivo* but also populates the satellite cell compartment in the host muscle.

Given the long-term persistence of human myofibers in *mdx* nude mice, we hypothesize that the human myofibers might be supported by innervation and vascularization in the host muscle tissue. By using fluorescence-conjugated α-Bungarotoxin to label the post-synaptic acetylcholine receptor (AChR) and antibodies against the pre-synaptic marker synaptic vesicle glycoprotein 2A (SV2), we detected not only AChR clustering on hDystrophin+ myofibers (Figure 5C) but also the co-localization of AChR and SV2 indicating the formation of neuromuscular junctions (NMJs) between mouse motor neuron and human myofibers (Figure 5C). Furthermore, using CD31 antibodies that recognize endothelial cells, we identified blood vessels spreading across the engrafted region of hDystrophin+ myofibers (Figure 5D),

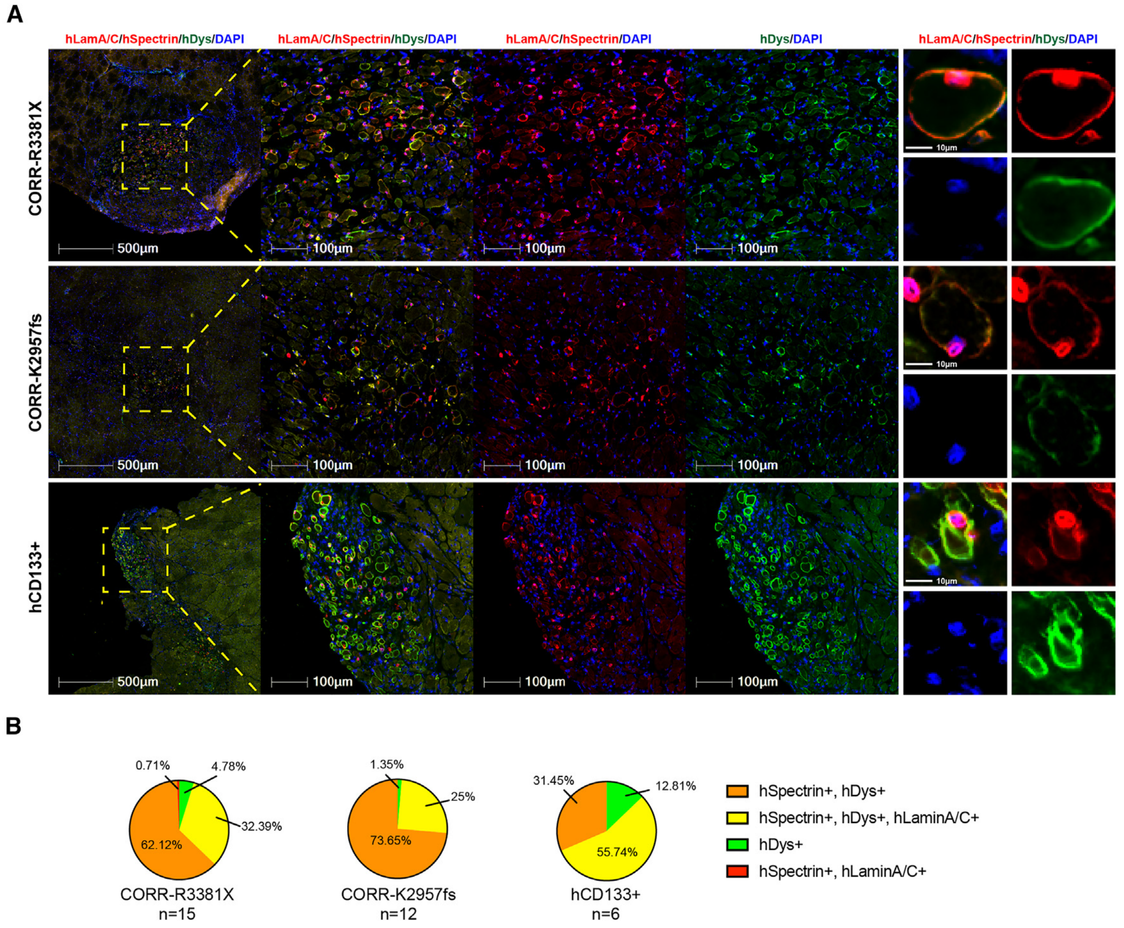

Figure 4. Distribution patterns of donor-derived human myofibers in *mdx* nude mice at 4 weeks after transplantation

(A) Representative transverse cryosections of donor-derived human myofibers derived from cell-laden constructs of CORR-R3381X MPCs or CORR-K2957fs MPCs in Promocell growth medium and hCD133+ cells in Megacell growth medium. Human lamin A/C and human spectrin (both red) and human dystrophin (green). Nuclei were counterstained with DAPI (blue). Scale bars, 10–500 μm as indicated in each panel.

(B) Pie charts show percentages of distribution patterns of donor-derived human myofibers in *mdx* nude mice. CORR-R3381X MPCs in Promocell growth medium (15 TA muscles), CORR-K2957fs MPCs in Promocell growth medium (12 TA muscles), and hCD133+ cells in Megacell growth medium (6 TA muscles).

supporting our hypothesis. Taken together, these results suggest that transplantation of 3D cell-laden hydrogel constructs supports long-term engraftment of human myofibers that are innervated and vascularized, together with PAX7+ cells of human origin populating satellite cell niche, in *mdx* nude mice.

Further maturation of CORR-R3381X MPC-derived human myofibers upon long-term engraftment

To investigate whether the sizes of donor-derived human myofibers might depend on cell sources and the time span of engraftment, we decided to compare three experimental conditions (CORR-R3381X, 4 weeks; hCD133+, 4 weeks; CORR-R3381X, 5 and 6 months) by measuring cross-section areas (CSAs) of human myofibers in 3 independent mouse TA muscles that gave the highest engraftment efficiency in each condition. Collectively, we recorded 391 CSAs in 3 CORR-R3381X 4-week TA muscles (median 317.2 μm^2 [181.6–494.7]), 518 CSAs in 3 hCD133+ 4-week TA muscles (median 172.5 μm^2 [101.0–302.6], and 185 CSAs in 3 CORR-R3381X 5- and 6-month TA muscles (median 344.5 μm^2 [174.5–577.5] (Table S3). Paradoxically, although the highest engraftment efficiency was achieved by hCD133+ cells, the overall CSAs of human myofibers derived from this cell type were significantly smaller than those of CORR-R3381X MPC-derived myofibers at 4 weeks or 5 and 6 months (Figure 6A).

Next, we sought to examine the relative frequency of myofiber CSAs in each experimental condition by plotting frequency distribution (Figure 6B; Table S4). We found that >44.59% of hCD133+ cell-derived myofiber CSAs were below 150 μm^2, while CORR-R3381X MPC-derived myofibers at 4 weeks or 5 and 6 months have <17.83% in the same category. All three conditions have similar relative frequencies of myofiber CSAs in the 150–350 μm^2 category, ranging from 32.97% to 40.15%. Among larger myofiber CSAs (>550 μm^2), the relative frequencies of CORR-R3381X MPC-derived myofibers at 4 weeks or 5 and 6 months were 18.41% and 27.56%, respectively. By fitting

CellPress
OPEN ACCESS

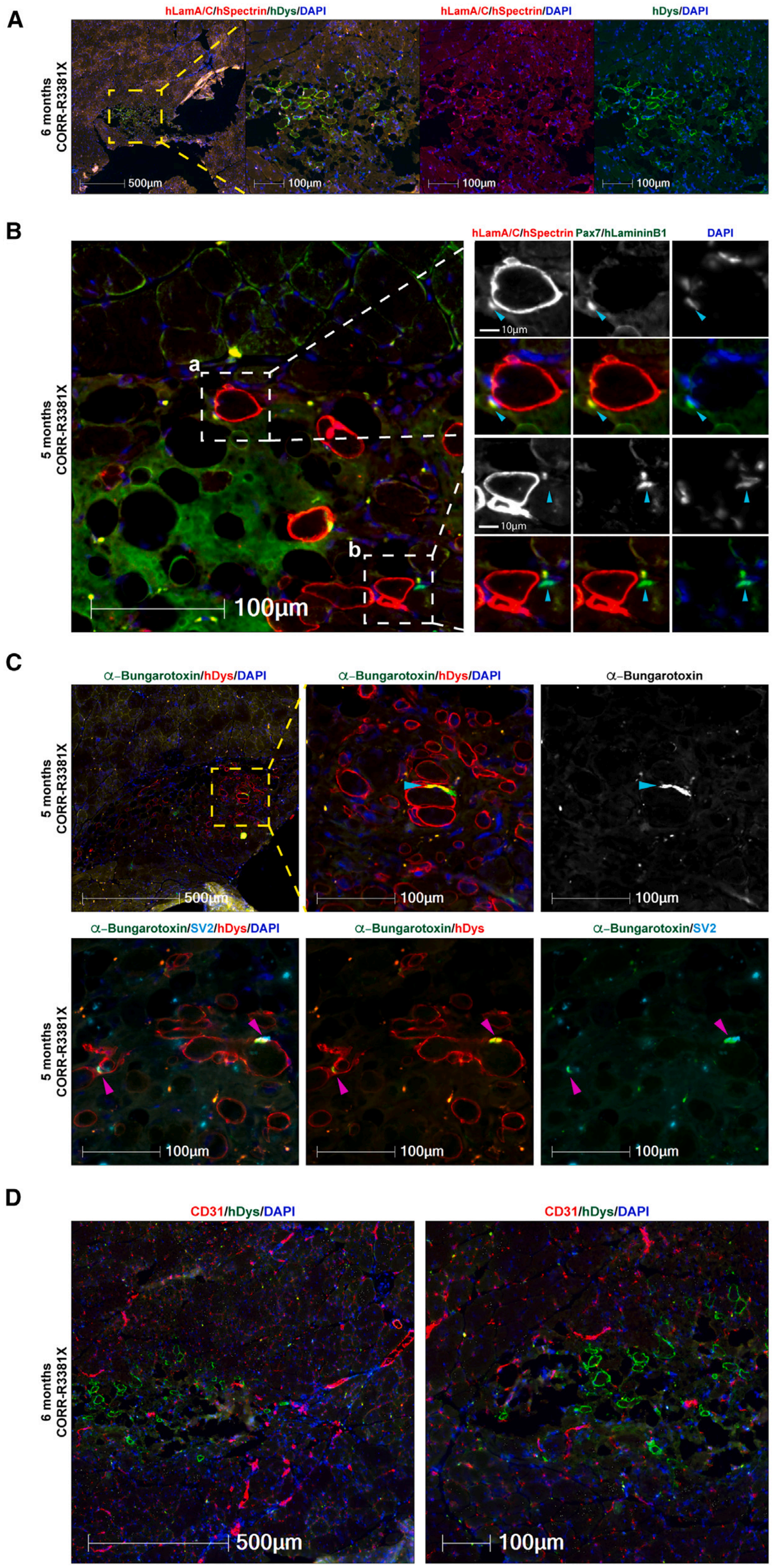

Figure 5. Long-term engraftment of human myofibers and human PAX7+ cells in the satellite cell niche with innervation and vascularization in *mdx* nude mice

(A) A representative transverse cryosection of *mdx* nude mouse TA muscle transplanted with a 3D construct of CORR-R3381X MPCs in Promocell growth medium (6 months after transplantation). Human lamin A/C and human spectrin (both red) and human dystrophin (green). Nuclei were labeled with DAPI (blue).

(B) Detection of human (a) and mouse (b) PAX7+ cells (arrowheads) in the satellite cell compartment at 5 months after transplantation. (a) A PAX7+ cell (green) of human origin labeled by human laminA/C (red) and DAPI (blue) adjacent to a human myofiber (hSpectrin, red) beneath the basal lamina (hLaminin β1, green). (b) A PAX7+ cell (green) of mouse origin with DAPI (blue) adjacent to a human myofiber (hSpectrin, red) within the basal lamina (hLaminin β1, green).

(C) Innervation of donor-derived human myofibers (hDystrophin, red) in *mdx* nude mice at 5 months after transplantation. The formation of NMJs (arrowheads) is demonstrated by co-localization of post-synaptic marker AChR (labeled by α-Bungarotoxin, green) and pre-synaptic marker SV2 (cyan). Nuclei were labeled with DAPI (blue).

(D) Vascularization in the engrafted regions at 6 months after transplantation as demonstrated by detection of blood vessels (CD31, red) adjacent to human myofibers (hDystrophin, green). Nuclei were labeled with DAPI (blue). 6 biological replicates for long-term engraftment experiment. Scale bars, 10–500 μm as indicated in each panel.

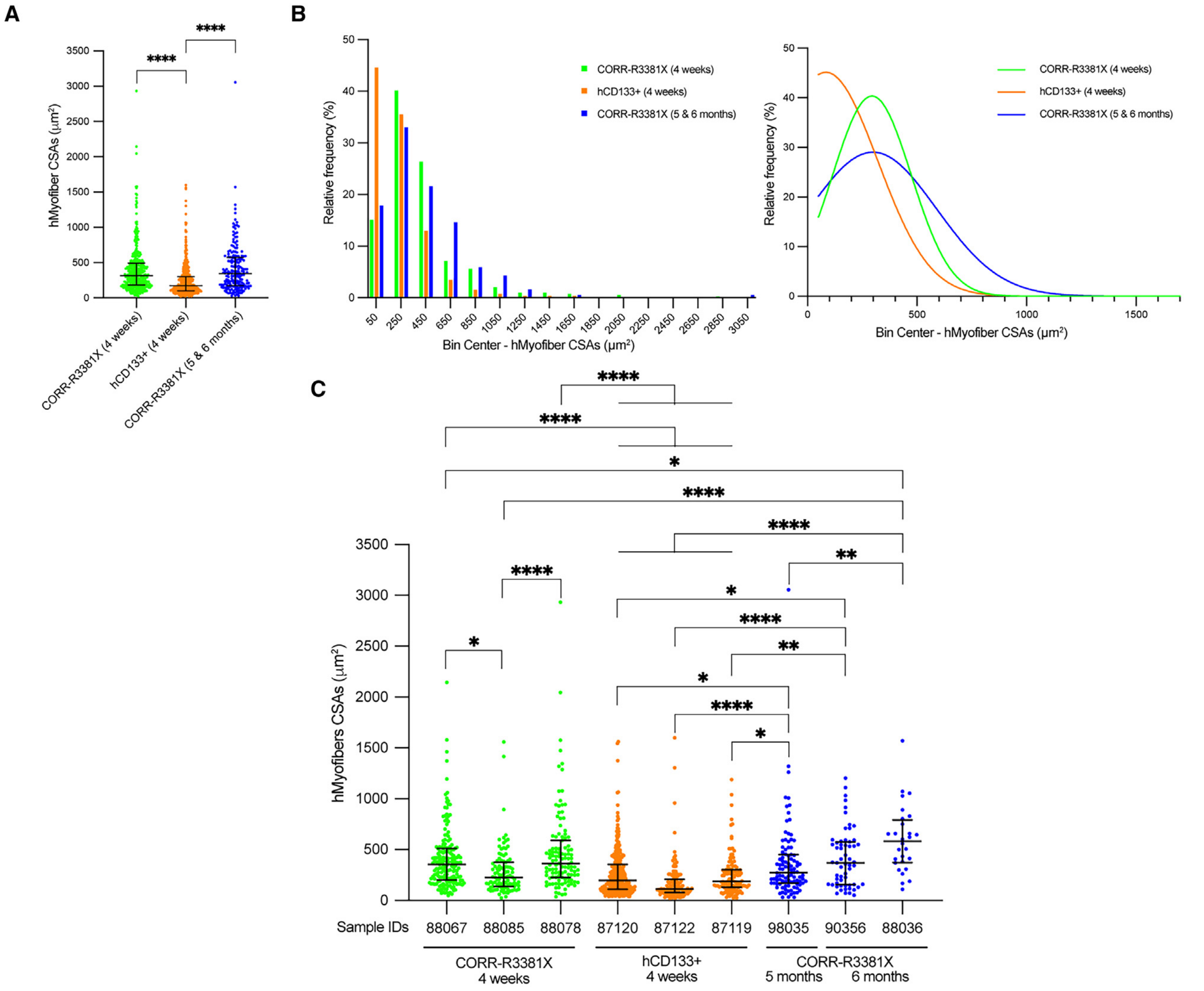

Figure 6. CORR-R3381X MPC-derived human myofibers matured further upon long-term engraftment

(A) Comparison of cross-section areas (CSAs) of donor-derived hMyofibers in mouse TA muscles from three experimental conditions, CORR-R3381X 4 weeks (391 CSAs from 3 samples), hCD133+ 4 weeks (518 CSAs from 3 samples), and CORR-R3381X 5 and 6 months (185 CSAs from 3 samples). Values indicate median (25th–75th percentile). One-way ANOVA and Tukey's multiple comparisons test; ****$p < 0.0001$.

(B) Relative frequency distribution (%) of CSAs of hMyofibers derived from CORR-R3381X MPCs (4 weeks), hCD133+ cells (4 weeks), and CORR-R3381X MPCs (5 and 6 months), which were then fitted with non-linear regression curves to indicate the trends in each condition.

(C) Comparisons of CSA measurements from individual TA muscle samples that ranked in top 3 engraftment efficiencies in each experimental condition. Values indicate median (25th–75th percentile). One-way ANOVA and Tukey's multiple comparisons test; *$p < 0.05$; **$p < 0.01$; ****$p < 0.0001$.

non-linear regression curves, we found that the relative frequencies of CORR-R3381X myofiber CSAs at 5 and 6 months shift further toward the right compared to those at 4 weeks (Figure 6B), suggesting that the sizes of CORR-R3381X myofiber became larger upon long-term engraftment.

To further distinguish differences of donor-derived human myofiber CSAs between individual mouse TA muscles, we then plotted CSA measurements per TA muscle sample separately (Figure 6C). We found that the CSAs of CORR-R3381X MPC-derived myofibers at 6 months (sample ID 88036; median 580.75 μm^2) were significantly larger than CSAs of other samples, although the differences were not statistically significant with sample ID 90356 (CORR-R3381X, 6 months; median 368.31 μm^2) and ID 88078 (CORR-R3381X, 4 weeks; median 364.3 μm^2). In addition, CSAs of sample ID 90356 (CORR-R3381X, 6 months) and ID 98035 (CORR-R3381X, 5 months), as well as sample ID 88067 and 88078 (CORR-R3381X, 4 weeks), were significantly larger than CSAs of sample ID 87120, 87122, or 87119 (hCD133+, 4 weeks). Altogether, our results suggest that sources of human myogenic cells may determine the overall sizes of myofibers upon engraftment. In addition, the fact that CORR-R3381X MPC-derived myofibers at 6 months were significantly larger than those at 4 weeks suggests that donor-derived human myofibers, supported by

innervation and vascularization, did not undergo atrophy and may have matured further upon long-term engraftment.

DISCUSSION

In this study, we have established a clinically relevant transplantation strategy that uses hydrogel-mediated deliveries of engineered human myogenic cells without modulation of the host muscles to achieve xenoengraftment in dystrophin-deficient *mdx* nude mice. Our transplantation procedure therefore improves animal welfare (3Rs impact: refinement, reduction, replacement). We have shown that two independent lines of CRISPR-corrected human PSC-derived MPCs and skeletal muscle-derived hCD133+ cells contribute to *in vivo* muscle regeneration with expression of full-length dystrophin. In addition, we have demonstrated innervation and vascularization of donor-derived human myofibers within *mdx* nude mouse muscles at 5–6 months after transplantation. Moreover, engrafted human myogenic cells gave rise to PAX7+ cells to populate the satellite cell niche. Importantly, there was no evidence for tumorigenesis in transplanted mice. Together, our results suggest that human PSC-derived MPCs are safe and stable for long-term engraftment. Transplantation of autologous MPCs rather than allogenic donor-derived MPCs reduces risk of the immunological response leading to rejection and reduces necessity for lifelong immunosuppression. Our findings suggest that it is possible to use CRISPR-corrected PSCs to generate MPCs to develop hydrogel-based cell therapy, and our strategy can be applied to other types of muscular dystrophy.[55] But our study also shows a wide variation of engraftment efficiencies between sources of myogenic cells, between individual host mice, and between transplantation experiments. Therefore, there is still room for further improvement to reduce the variability and increase the engraftment efficiency.

Several factors may affect the variability of engraftment efficiency. For instance, quantification of myofibers of human origin could be affected by technical aspects related to the experimental and analysis methods. In 10 μm sections of analyzed host *mdx* nude TA muscles, we could not always detect nuclei in all the donor-derived human myofibers. In addition, we used a stringent quantification method, which excluded hSpectrin+ only myofibers as human origin if they did not have hLaminA/C+ nuclei nor hDystrophin+ immunofluorescence. Thus, our study is likely to underestimate myofibers of human origin. Another pertinent factor is that transplanted hydrogel constructs may have been squeezed either to the edge or outside the grafted muscle, which might affect the engraftment efficiency. After immunofluorescence analysis with human-specific antibodies, we observed 32.14% of transplants at the edge of the host muscle, which indicate sub-optimal hydrogel placement, resulting in technical issues with cryosectioning and further analysis. For example, we observed that during cryosectioning the outer part of the muscle section occasionally detached from the rest, which could also have a negative impact on our quantification. Notably, even though nude mice are T cell deficient, they maintain B cell activity and high natural killer (NK) cell function.[56,57] In this respect, low number of donor-derived human myofibers in *mdx* nude mice might be because of xenografts suffering some immunological rejection in host mice, which are not completely immunodeficient. Moreover, previous data suggested that genetic background has an influence on *mdx* mouse muscle regeneration.[58] Therefore, differences of genetic backgrounds between our *mdx* nude mice and other groups' dystrophin-deficient immunodeficient host mice might also affect the number of myofibers of human origin in different studies.

Apart from host animal strains,[19] modulation methods of host muscle may also affect the engraftment efficiency. When transplanting human PSC-derived myogenic cells into barium chloride-treated NSG host muscles, there were 35–58 donor-derived myofibers,[59] and, following transplant into cardiotoxin-treated NSG host muscles, there were approximately 20–60 myofibers of donor origin[60] or 96–190 myofibers of donor origin in cardiotoxin-treated mdx-NSG host muscles.[46] The results of our present study are in the range of the results of these studies. Prior to transplantation of donor cells, host muscles can be irradiated, cryoinjured, or both.[19] Irradiation has been used to recapitulate the DMD pathology in *mdx* mice, in which satellite cell exhaustion hinders muscle regeneration.[61] In addition, irradiation spares myofibers and preserves the muscle stem cell niche and has an influence on the regenerative potential of transplanted cells.[18] Indeed, previous studies showed that significantly more donor-derived dystrophin+ myofibers were present in irradiated than in non-irradiated host muscles after immortalized mouse myoblasts[62] or mouse satellite cells[5,18,63] were transplanted into *mdx* nude mice, but host muscle irradiation did not augment muscle regeneration derived from human muscle precursor cells.[19] Notably, childhood cancer survivors have shown that radiation exposure in early life stage can cause radiation-induced fibrosis affecting 80% of patients,[64] leading to muscle atrophy, impaired mobility, and weakness.[65] Thus, it is desirable to develop new clinically safe approaches boosting donor cell engraftment in future cell therapies for DMD patients.

Previous studies have shown regenerative potential of mouse[6,18] or human[66] satellite cells after transplantation into host mouse muscles. These satellite cells demonstrated engraftment and generation of myofibers of donor origin within the host muscles. It has been also shown that intramuscular transplantation of human postnatal myoblasts, skeletal muscle-derived hCD133+ cells, or human PSC-derived MPCs into immunodeficient mice can give rise to functional satellite cells of donor origin.[11,16,67] We hypothesize that PAX7+ cells of human origin after transplantation in our study may be functionally equivalent to satellite cells. However, this will require further investigation to demonstrate whether these PAX7+ cells of human origin in the satellite cell niche are able to contribute to host muscle regeneration after re-injury.

In conclusion, we have shown that hydrogel-mediated delivery is suitable for long-term engraftment of CRISPR-corrected human PSC-derived MPCs in unmodulated *mdx* nude mouse muscles, enabling full-length dystrophin expression, innervation, and vascularization in the engrafted region and populating the satellite cell niche. In this regard, our study therefore presents a clinically relevant transplantation strategy. Advances in xeno-free natural or synthetic biomaterials that can replace Matrigel-based hydrogels and support regenerative potential of human MPCs will pave ways for clinical trials of hydrogel-mediated cell

therapy. Future improvement in engraftment efficiency with evidence of improved muscle function may lead to therapeutic application for muscular dystrophies.

Limitations of the study

Firstly, our fibrin hydrogel is supplemented with Matrigel for cell encapsulation to generate hydrogel constructs. Since Matrigel is a solubilized basement membrane preparation derived from the Engelbreth-Holm-Swarm mouse sarcoma, many inherent issues are associated with Matrigel, such as lot-to-lot variation of extracellular matrix composition, potential pathogen transmission, and risks for immunogenicity in humans. In comparison with a recent study by Wu et al.,[68] a fibrin hydrogel (without Matrigel) was used to cast human PSC-derived myogenic cells *in situ* in an NSG mouse model of volumetric muscle loss, providing evidence of ~60 human/~80 hybrid myofibers with human PAX7+ cells, innervation, and vascularization at 5 weeks after the treatment. According to the study by Wu et al., if we remove Matrigel in our delivery system, we expect significantly reduced xenoengraftment as our host environment is dystrophic and T cells (−)/NK cells (+). This highlights the need to develop xeno-free natural hydrogels (e.g., protein polymers and decellularized extracellular matrix) or synthetic biomaterials (e.g., polyethylene glycol macromer)[25,26] suitable for hydrogel-mediated delivery of human MPCs with improved engraftment efficiency. Our present proof-of-concept study provides an important benchmark for evaluating new biocompatible hydrogels that are currently under development by others in future xenotransplantation studies. Secondly, in our study, the percentage of donor-derived human myofibers per mouse TA muscle (~2,000 myofibers) is lower than 20% in any set of transplantations. Although it seems that only modest levels of dystrophin are required for functional benefit,[69–74] to be fully protective, dystrophin at low levels must spread all along the myofiber[75] and be present in most of the fibers within a muscle. However, the contribution of donor cell-derived dystrophin to host mouse muscle is segmental,[53,54,76] and the spreading of dystrophin is only a few hundred microns from the nucleus producing it.[53] For these reasons, we did not perform experiments to assess functional improvements in *mdx* nude TA muscles transplanted with CORR-R3381X or CORR-K2957fs cell-laden constructs. Nevertheless, when future studies achieve 10%–20% of dystrophin present in the majority of myofibers within the treated muscle, it will be of interest to assess whether xenoengraftment is able to improve muscle function of dystrophin-deficient host mice.

RESOURCE AVAILABILITY

Lead contact

Further information and requests will be fulfilled by the lead contact, Yung-Yao Lin (yy.lin@qmul.ac.uk).

Materials availability

This study did not generate new unique reagents.

Data and code availability

Bulk RNA-seq data were deposited in the Gene Expression Omnibus (accession numbers GSE159273 and GSE189053).[33,34] This paper does not report original code. All non-protected additional data are available from the lead contact upon request.

ACKNOWLEDGMENTS

We thank Penney Gilbert, Majid Ebrahimi, and Ratima Suntornnond for helping in fabrication of PDMS molds; William Weston for assistance in H&E staining; Luke Gammon for help with Cell DIVE imaging; and Servier Medical Art for some figure elements under a Creative Commons Attribution 3.0 Unported Licence. This research was primarily funded by the Barts Charity grant MGU0426 to Y.-Y.L. and J.E.M. A.K. was funded by the QMUL-Life Sciences Initiative PhD studentship and the Barts Charity. This work was in part supported by the Royal Society grant RG130417, Newlife the Charity for Disabled Children grant SG/14-15/14, Action Duchenne grant AD1801Y, Duchenne Parent Project grant 19.017, and Queen Mary Impact Fund to Y.-Y.L. The support of National Centre for the Replacement, Refinement and Reduction of Animals in Research (NC3Rs) grants NC/T002085/1 and NC/C020109/1 to Y.-Y.L. is also gratefully acknowledged.

AUTHOR CONTRIBUTIONS

A.K. conducted most of the experimental work and analyzed and interpreted the data. J.B. performed the transcriptome analysis. J.M. and J.E.M. performed some of the experiments. J.E.M. and Y.-Y.L. designed the experiments, analyzed and interpreted the data, and supervised the research. C.A.M., O.P., and J.C. provided essential expertise, materials, and technical support for the experiments. A.K., J.E.M., and Y.-Y.L. co-wrote the manuscript with input from all authors.

DECLARATION OF INTERESTS

Y.-Y.L. was the Principal Investigator in a research project funded by Pfizer. O.P. is a co-founder and shareholder of Somite Therapeutics.

STAR★METHODS

Detailed methods are provided in the online version of this paper and include the following:

- KEY RESOURCES TABLE
- EXPERIMENTAL MODEL AND STUDY PARTICIPANT DETAILS
  - Animal model
  - Human myogenic cells
  - hCD133+ cells
  - Human PSC-derived MPCs
- METHOD DETAILS
  - Transcriptome analysis
  - Manufacturing PDMS molds
  - Cell encapsulation in hydrogel within PDMS molds
  - *In vivo* transplantation procedure
  - TA muscle tissue processing
  - Immunocytochemistry
  - Histological staining
- QUANTIFICATION AND STATISTICAL ANALYSIS

SUPPLEMENTAL INFORMATION

Supplemental information can be found online at https://doi.org/10.1016/j.xcrm.2025.102019.

Received: November 10, 2023
Revised: July 29, 2024
Accepted: February 17, 2025
Published: March 7, 2025

REFERENCES

1. Mauro, A. (1961). SATELLITE CELL OF SKELETAL MUSCLE FIBERS. J. Biophys. Biochem. Cytol. *9*, 493–495. https://doi.org/10.1083/jcb.9.2.493.

2. Montarras, D., Morgan, J., Collins, C., Relaix, F., Zaffran, S., Cumano, A., Partridge, T., and Buckingham, M. (2005). Direct isolation of satellite cells for skeletal muscle regeneration. Science *309*, 2064–2067. https://doi.org/10.1126/science.1114758.

3. Yin, H., Price, F., and Rudnicki, M.A. (2013). Satellite Cells and the Muscle Stem Cell Niche. Physiol. Rev. *93*, 23–67. https://doi.org/10.1152/physrev.00043.2011.

4. Mercuri, E., Bönnemann, C.G., and Muntoni, F. (2019). Muscular dystrophies. Lancet *394*, 2025–2038. https://doi.org/10.1016/S0140-6736(19)32910-1.

5. Boldrin, L., Zammit, P.S., and Morgan, J.E. (2015). Satellite cells from dystrophic muscle retain regenerative capacity. Stem Cell Res. *14*, 20–29. https://doi.org/10.1016/j.scr.2014.10.007.

6. Collins, C.A., Olsen, I., Zammit, P.S., Heslop, L., Petrie, A., Partridge, T.A., and Morgan, J.E. (2005). Stem Cell Function, Self-Renewal, and Behavioral Heterogeneity of Cells from the Adult Muscle Satellite Cell Niche. Cell *122*, 289–301. https://doi.org/10.1016/j.cell.2005.05.010.

7. Karpati, G., Pouliot, Y., Zubrzycka-Gaarn, E., Carpenter, S., Ray, P.N., Worton, R.G., and Holland, P. (1989). Dystrophin is expressed in mdx skeletal muscle fibers after normal myoblast implantation. Am. J. Pathol. *135*, 27–32.

8. Partridge, T.A., Morgan, J.E., Coulton, G.R., Hoffman, E.P., and Kunkel, L.M. (1989). Conversion of mdx myofibres from dystrophin-negative to -positive by injection of normal myoblasts. Nature *337*, 176–179. https://doi.org/10.1038/337176a0.

9. Dellavalle, A., Sampaolesi, M., Tonlorenzi, R., Tagliafico, E., Sacchetti, B., Perani, L., Innocenzi, A., Galvez, B.G., Messina, G., Morosetti, R., et al. (2007). Pericytes of human skeletal muscle are myogenic precursors distinct from satellite cells. Nat. Cell Biol. *9*, 255–267. https://doi.org/10.1038/ncb1542.

10. Benchaouir, R., Meregalli, M., Farini, A., D'Antona, G., Belicchi, M., Goyenvalle, A., Battistelli, M., Bresolin, N., Bottinelli, R., Garcia, L., and Torrente, Y. (2007). Restoration of Human Dystrophin Following Transplantation of Exon-Skipping-Engineered DMD Patient Stem Cells into Dystrophic Mice. Cell Stem Cell *1*, 646–657. https://doi.org/10.1016/j.stem.2007.09.016.

11. Meng, J., Chun, S., Asfahani, R., Lochmüller, H., Muntoni, F., and Morgan, J. (2014). Human Skeletal Muscle–derived CD133+ Cells Form Functional Satellite Cells After Intramuscular Transplantation in Immunodeficient Host Mice. Mol. Ther. *22*, 1008–1017. https://doi.org/10.1038/mt.2014.26.

12. Sampaolesi, M., Blot, S., D'Antona, G., Granger, N., Tonlorenzi, R., Innocenzi, A., Mognol, P., Thibaud, J.-L., Galvez, B.G., Barthélémy, I., et al. (2006). Mesoangioblast stem cells ameliorate muscle function in dystrophic dogs. Nature *444*, 574–579. https://doi.org/10.1038/nature05282.

13. Paredes-Redondo, A., and Lin, Y. (2019). Human Induced Pluripotent Stem Cells: Challenges and Opportunities in Developing New Therapies for Muscular Dystrophies. eLS, 1–10. https://doi.org/10.1002/9780470015902.a0028371.

14. Yan, L., Rodríguez-delaRosa, A., and Pourquié, O. (2021). Human muscle production in vitro from pluripotent stem cells: Basic and clinical applications. Semin. Cell Dev. Biol. *119*, 39–48. https://doi.org/10.1016/j.semcdb.2021.04.017.

15. Al Tanoury, Z., Rao, J., Tassy, O., Gobert, B., Gapon, S., Garnier, J.-M., Wagner, E., Hick, A., Hall, A., Gussoni, E., and Pourquié, O. (2020). Differentiation of the human PAX7-positive myogenic precursors/satellite cell lineage in vitro. Development *147*, dev187344. https://doi.org/10.1242/dev.187344.

16. Sun, C., Kannan, S., Choi, I.Y., Lim, H., Zhang, H., Chen, G.S., Zhang, N., Park, S.-H., Serra, C., Iyer, S.R., et al. (2022). Human pluripotent stem cell-derived myogenic progenitors undergo maturation to quiescent satellite cells upon engraftment. Cell Stem Cell *29*, 610–619.e5. https://doi.org/10.1016/j.stem.2022.03.004.

17. Darabi, R., Arpke, R.W., Irion, S., Dimos, J.T., Grskovic, M., Kyba, M., and Perlingeiro, R.C.R. (2012). Human ES- and iPS-Derived Myogenic Progenitors Restore DYSTROPHIN and Improve Contractility upon Transplantation in Dystrophic Mice. Cell Stem Cell *10*, 610–619. https://doi.org/10.1016/j.stem.2012.02.015.

18. Boldrin, L., Neal, A., Zammit, P.S., Muntoni, F., and Morgan, J.E. (2012). Donor Satellite Cell Engraftment Is Significantly Augmented When the Host Niche Is Preserved and Endogenous Satellite Cells Are Incapacitated. Stem Cell. *30*, 1971–1984. https://doi.org/10.1002/stem.1158.

19. Meng, J., Bencze, M., Asfahani, R., Muntoni, F., and Morgan, J.E. (2015). The effect of the muscle environment on the regenerative capacity of human skeletal muscle stem cells. Skeletal Muscle *5*, 11. https://doi.org/10.1186/s13395-015-0036-8.

20. Gussoni, E., Blau, H.M., and Kunkel, L.M. (1997). The fate of individual myoblasts after transplantation into muscles of DMD patients. Nat. Med. *3*, 970–977. https://doi.org/10.1038/nm0997-970.

21. Mendell, J.R., Kissel, J.T., Amato, A.A., King, W., Signore, L., Prior, T.W., Sahenk, Z., Benson, S., McAndrew, P.E., Rice, R., et al. (1995). Myoblast Transfer in the Treatment of Duchenne's Muscular Dystrophy. N. Engl. J. Med. *333*, 832–838. https://doi.org/10.1056/NEJM199509283331303.

22. Skuk, D., Roy, B., Goulet, M., and Tremblay, J.P. (1999). Successful myoblast transplantation in primates depends on appropriate cell delivery and induction of regeneration in the host muscle. Exp. Neurol. *155*, 22–30. https://doi.org/10.1006/exnr.1998.6973.

23. Skuk, D., Goulet, M., and Tremblay, J.P. (2014). Intramuscular transplantation of myogenic cells in primates: importance of needle size, cell number, and injection volume. Cell Transplant. *23*, 13–25. https://doi.org/10.3727/096368912X661337.

24. Afshar Bakooshli, M., Lippmann, E.S., Mulcahy, B., Iyer, N., Nguyen, C.T., Tung, K., Stewart, B.A., van den Dorpel, H., Fuehrmann, T., Shoichet, M., et al. (2019). A 3D culture model of innervated human skeletal muscle enables studies of the adult neuromuscular junction. Elife *8*, e44530. https://doi.org/10.7554/eLife.44530.

25. Han, W.M., Anderson, S.E., Mohiuddin, M., Barros, D., Nakhai, S.A., Shin, E., Amaral, I.F., Pêgo, A.P., García, A.J., and Jang, Y.C. (2018). Synthetic matrix enhances transplanted satellite cell engraftment in dystrophic and aged skeletal muscle with comorbid trauma. Sci. Adv. *4*, eaar4008. https://doi.org/10.1126/sciadv.aar4008.

26. Lev, R., and Seliktar, D. (2018). Hydrogel biomaterials and their therapeutic potential for muscle injuries and muscular dystrophies. J. R. Soc. Interface *15*, 20170380. https://doi.org/10.1098/rsif.2017.0380.

27. Maffioletti, S.M., Sarcar, S., Henderson, A.B.H., Mannhardt, I., Pinton, L., Moyle, L.A., Steele-Stallard, H., Cappellari, O., Wells, K.E., Ferrari, G., et al. (2018). Three-Dimensional Human iPSC-Derived Artificial Skeletal Muscles Model Muscular Dystrophies and Enable Multilineage Tissue Engineering. Cell Rep. *23*, 899–908. https://doi.org/10.1016/j.celrep.2018.03.091.

28. Rao, L., Qian, Y., Khodabukus, A., Ribar, T., and Bursac, N. (2018). Engineering human pluripotent stem cells into a functional skeletal muscle tissue. Nat. Commun. *9*, 126. https://doi.org/10.1038/s41467-017-02636-4.

29. Quarta, M., Cromie, M., Chacon, R., Blonigan, J., Garcia, V., Akimenko, I., Hamer, M., Paine, P., Stok, M., Shrager, J.B., and Rando, T.A. (2017). Bioengineered constructs combined with exercise enhance stem cell-mediated treatment of volumetric muscle loss. Nat. Commun. *8*, 15613. https://doi.org/10.1038/ncomms15613.

30. Fuoco, C., Rizzi, R., Biondo, A., Longa, E., Mascaro, A., Shapira-Schweitzer, K., Kossovar, O., Benedetti, S., Salvatori, M.L., Santoleri, S., et al. (2015). In vivo generation of a mature and functional artificial skeletal muscle. EMBO Mol. Med. *7*, 411–422. https://doi.org/10.15252/emmm.201404062.

31. Kim, J.H., Seol, Y.-J., Ko, I.K., Kang, H.-W., Lee, Y.K., Yoo, J.J., Atala, A., and Lee, S.J. (2018). 3D Bioprinted Human Skeletal Muscle Constructs for Muscle Function Restoration. Sci. Rep. *8*, 12307. https://doi.org/10.1038/s41598-018-29968-5.

32. Kim, J.H., Kim, I., Seol, Y.-J., Ko, I.K., Yoo, J.J., Atala, A., and Lee, S.J. (2020). Neural cell integration into 3D bioprinted skeletal muscle constructs accelerates restoration of muscle function. Nat. Commun. *11*, 1025. https://doi.org/10.1038/s41467-020-14930-9.

33. Paredes-Redondo, A., Harley, P., Maniati, E., Ryan, D., Louzada, S., Meng, J., Kowala, A., Fu, B., Yang, F., Liu, P., et al. (2021). Optogenetic modeling of human neuromuscular circuits in Duchenne muscular dystrophy with CRISPR and pharmacological corrections. Sci. Adv. *7*, eabi8787. https://doi.org/10.1126/sciadv.abi8787.

34. Morera, C., Kim, J., Paredes-Redondo, A., Nobles, M., Rybin, D., Moccia, R., Kowala, A., Meng, J., Garren, S., Liu, P., et al. (2022). CRISPR-mediated correction of skeletal muscle Ca2+ handling in a novel DMD patient-derived pluripotent stem cell model. Neuromuscul. Disord. *32*, 908–922. https://doi.org/10.1016/j.nmd.2022.10.007.

35. Dobin, A., Davis, C.A., Schlesinger, F., Drenkow, J., Zaleski, C., Jha, S., Batut, P., Chaisson, M., and Gingeras, T.R. (2013). STAR: ultrafast universal RNA-seq aligner. Bioinformatics *29*, 15–21. https://doi.org/10.1093/bioinformatics/bts635.

36. Kolberg, L., Raudvere, U., Kuzmin, I., Adler, P., Vilo, J., and Peterson, H. (2023). g:Profiler-interoperable web service for functional enrichment analysis and gene identifier mapping (2023 update). Nucleic Acids Res. *51*, W207–W212. https://doi.org/10.1093/nar/gkad347.

37. Sousa-Victor, P., García-Prat, L., and Muñoz-Cánoves, P. (2022). Control of satellite cell function in muscle regeneration and its disruption in ageing. Nat. Rev. Mol. Cell Biol. *23*, 204–226. https://doi.org/10.1038/s41580-021-00421-2.

38. Cosgrove, B.D., Gilbert, P.M., Porpiglia, E., Mourkioti, F., Lee, S.P., Corbel, S.Y., Llewellyn, M.E., Delp, S.L., and Blau, H.M. (2014). Rejuvenation of the muscle stem cell population restores strength to injured aged muscles. Nat. Med. *20*, 255–264. https://doi.org/10.1038/nm.3464.

39. Bernet, J.D., Doles, J.D., Hall, J.K., Kelly Tanaka, K., Carter, T.A., and Olwin, B.B. (2014). p38 MAPK signaling underlies a cell-autonomous loss of stem cell self-renewal in skeletal muscle of aged mice. Nat. Med. *20*, 265–271. https://doi.org/10.1038/nm.3465.

40. Perdiguero, E., Ruiz-Bonilla, V., Serrano, A.L., and Muñoz-Cánoves, P. (2007). Genetic deficiency of p38alpha reveals its critical role in myoblast cell cycle exit: the p38alpha-JNK connection. Cell Cycle *6*, 1298–1303. https://doi.org/10.4161/cc.6.11.4315.

41. von Maltzahn, J., Chang, N.C., Bentzinger, C.F., and Rudnicki, M.A. (2012). Wnt signaling in myogenesis. Trends Cell Biol. *22*, 602–609. https://doi.org/10.1016/j.tcb.2012.07.008.

42. Conboy, I.M., Conboy, M.J., Smythe, G.M., and Rando, T.A. (2003). Notch-mediated restoration of regenerative potential to aged muscle. Science *302*, 1575–1577. https://doi.org/10.1126/science.1087573.

43. Dumont, N.A., Wang, Y.X., Von Maltzahn, J., Pasut, A., Bentzinger, C.F., Brun, C.E., and Rudnicki, M.A. (2015). Dystrophin expression in muscle stem cells regulates their polarity and asymmetric division. Nat. Med. *21*, 1455–1463. https://doi.org/10.1038/nm.3990.

44. Subramanian, A., Tamayo, P., Mootha, V.K., Mukherjee, S., Ebert, B.L., Gillette, M.A., Paulovich, A., Pomeroy, S.L., Golub, T.R., Lander, E.S., and Mesirov, J.P. (2005). Gene set enrichment analysis: A knowledge-based approach for interpreting genome-wide expression profiles. Proc. Natl. Acad. Sci. USA *102*, 15545–15550. https://doi.org/10.1073/pnas.0506580102.

45. Bentzinger, C.F., Wang, Y.X., and Rudnicki, M.A. (2012). Building muscle: molecular regulation of myogenesis. Cold Spring Harbor Perspect. Biol. *4*, a008342. https://doi.org/10.1101/cshperspect.a008342.

46. Hicks, M.R., Hiserodt, J., Paras, K., Fujiwara, W., Eskin, A., Jan, M., Xi, H., Young, C.S., Evseenko, D., Nelson, S.F., et al. (2018). ERBB3 and NGFR mark a distinct skeletal muscle progenitor cell in human development and hPSCs. Nat. Cell Biol. *20*, 46–57. https://doi.org/10.1038/s41556-017-0010-2.

47. Shea, K.L., Xiang, W., LaPorta, V.S., Licht, J.D., Keller, C., Basson, M.A., and Brack, A.S. (2010). Sprouty1 regulates reversible quiescence of a self-renewing adult muscle stem cell pool during regeneration. Cell Stem Cell *6*, 117–129. https://doi.org/10.1016/j.stem.2009.12.015.

48. Segalés, J., Perdiguero, E., and Muñoz-Cánoves, P. (2016). Regulation of Muscle Stem Cell Functions: A Focus on the p38 MAPK Signaling Pathway. Front. Cell Dev. Biol. *4*, 91. https://doi.org/10.3389/fcell.2016.00091.

49. Chang, N.C., Sincennes, M.-C., Chevalier, F.P., Brun, C.E., Lacaria, M., Segalés, J., Muñoz-Cánoves, P., Ming, H., and Rudnicki, M.A. (2018). The Dystrophin Glycoprotein Complex Regulates the Epigenetic Activation of Muscle Stem Cell Commitment. Cell Stem Cell *22*, 755–768.e6. https://doi.org/10.1016/j.stem.2018.03.022.

50. Price, F.D., von Maltzahn, J., Bentzinger, C.F., Dumont, N.A., Yin, H., Chang, N.C., Wilson, D.H., Frenette, J., and Rudnicki, M.A. (2014). Inhibition of JAK-STAT signaling stimulates adult satellite cell function. Nat. Med. *20*, 1174–1181. https://doi.org/10.1038/nm.3655.

51. Sousa-Victor, P., Gutarra, S., García-Prat, L., Rodriguez-Ubreva, J., Ortet, L., Ruiz-Bonilla, V., Jardí, M., Ballestar, E., González, S., Serrano, A.L., et al. (2014). Geriatric muscle stem cells switch reversible quiescence into senescence. Nature *506*, 316–321. https://doi.org/10.1038/nature13013.

52. Rozkalne, A., Adkin, C., Meng, J., Lapan, A., Morgan, J.E., and Gussoni, E. (2014). Mouse regenerating myofibers detected as false-positive donor myofibers with anti-human spectrin. Hum. Gene Ther. *25*, 73–81. https://doi.org/10.1089/hum.2013.126.

53. Blaveri, K., Heslop, L., Yu, D.S., Rosenblatt, J.D., Gross, J.G., Partridge, T.A., and Morgan, J.E. (1999). Patterns of repair of dystrophic mouse muscle: studies on isolated fibers. Dev. Dyn. *216*, 244–256.

54. Brimah, K., Ehrhardt, J., Mouly, V., Butler-Browne, G.S., Partridge, T.A., and Morgan, J.E. (2004). Human Muscle Precursor Cell Regeneration in the Mouse Host Is Enhanced by Growth Factors. Hum. Gene Ther. *15*, 1109–1124. https://doi.org/10.1089/hum.2004.15.1109.

55. Kim, J., Lana, B., Torelli, S., Ryan, D., Catapano, F., Ala, P., Luft, C., Stevens, E., Konstantinidis, E., Louzada, S., et al. (2019). A new patient-derived iPSC model for dystroglycanopathies validates a compound that increases glycosylation of α-dystroglycan. EMBO Rep. *20*, e47967. https://doi.org/10.15252/embr.201947967.

56. Pelleitier, M., and Montplaisir, S. (1975). The nude mouse: a model of deficient T-cell function. Methods Achiev. Exp. Pathol. *7*, 149–166.

57. Budzynski, W., and Radzikowski, C. (1994). Cytotoxic Cs in immunodeficient athymic mice. Immunopharmacol. Immunotoxicol. *16*, 319–346. https://doi.org/10.3109/08923979409007097.

58. Fukada, S.i., Morikawa, D., Yamamoto, Y., Yoshida, T., Sumie, N., Yamaguchi, M., Ito, T., Miyagoe-Suzuki, Y., Takeda, S., Tsujikawa, K., and Yamamoto, H. (2010). Genetic background affects properties of satellite cells and mdx phenotypes. Am. J. Pathol. *176*, 2414–2424. https://doi.org/10.2353/ajpath.2010.090887.

59. van der Wal, E., Herrero-Hernandez, P., Wan, R., Broeders, M., van Gestel, T.J., van IJcken, W.F., Cheung, T.H., van der Ploeg, A.T., and Schaaf, G.J. (2018). Large-Scale Expansion of Human iPSC-Derived Skeletal Muscle Cells for Disease Modeling and Cell-Based Therapeutic Strategies. Stem Cell Rep. *10*, 1975–1990. https://doi.org/10.1016/j.stemcr.2018.04.002.

60. Zhao, M., Tazumi, A., Takayama, S., Takenaka-Ninagawa, N., Nalbandian, M., Nagai, M., Nakamura, Y., Nakasa, M., Watanabe, A., Ikeya, M., et al. (2020). Induced Fetal Human Muscle Stem Cells with High Therapeutic Potential in a Mouse Muscular Dystrophy Model. Stem Cell Rep. *15*, 80–94. https://doi.org/10.1016/j.stemcr.2020.06.004.

61. Wakeford, S., Watt, D.J., and Partridge, T.A. (1991). X-irradiation improves mdx mouse muscle as a model of myofiber loss in DMD. Muscle Nerve *14*, 42–50. https://doi.org/10.1002/mus.880140108.

62. Morgan, J.E., Gross, J.G., Pagel, C.N., Beauchamp, J.R., Fassati, A., Thrasher, A.J., Di Santo, J.P., Fisher, I.B., Shiwen, X., Abraham, D.J., and Partridge, T.A. (2002). Myogenic cell proliferation and generation of a reversible tumorigenic phenotype are triggered by preirradiation of the recipient site. J. Cell Biol. *157*, 693–702. https://doi.org/10.1083/jcb.200108047.
63. Doreste, B., Torelli, S., and Morgan, J. (2020). Irradiation dependent inflammatory response may enhance satellite cell engraftment. Sci. Rep. *10*, 11119. https://doi.org/10.1038/s41598-020-68098-9.
64. Paulino, A.C. (2004). Late effects of radiotherapy for pediatric extremity sarcomas. Int. J. Radiat. Oncol. Biol. Phys. *60*, 265–274. https://doi.org/10.1016/j.ijrobp.2004.02.001.
65. Stubblefield, M.D. (2011). Radiation Fibrosis Syndrome: Neuromuscular and Musculoskeletal Complications in Cancer Survivors. Pharm. Manag. PM R. *3*, 1041–1054. https://doi.org/10.1016/j.pmrj.2011.08.535.
66. Xu, X., Wilschut, K.J., Kouklis, G., Tian, H., Hesse, R., Garland, C., Sbitany, H., Hansen, S., Seth, R., Knott, P.D., et al. (2015). Human Satellite Cell Transplantation and Regeneration from Diverse Skeletal Muscles. Stem Cell Rep. *5*, 419–434. https://doi.org/10.1016/j.stemcr.2015.07.016.
67. Skuk, D., Paradis, M., Goulet, M., Chapdelaine, P., Rothstein, D.M., and Tremblay, J.P. (2010). Intramuscular Transplantation of Human Postnatal Myoblasts Generates Functional Donor-Derived Satellite Cells. Mol. Ther. *18*, 1689–1697. https://doi.org/10.1038/mt.2010.128.
68. Wu, J., Matthias, N., Bhalla, S., and Darabi, R. (2021). Evaluation of the Therapeutic Potential of Human iPSCs in a Murine Model of VML. Mol. Ther. *29*, 121–131. https://doi.org/10.1016/j.ymthe.2020.09.012.
69. Li, D., Yue, Y., and Duan, D. (2008). Preservation of muscle force in Mdx3cv mice correlates with low-level expression of a near full-length dystrophin protein. Am. J. Pathol. *172*, 1332–1341. https://doi.org/10.2353/ajpath.2008.071042.
70. Neri, M., Torelli, S., Brown, S., Ugo, I., Sabatelli, P., Merlini, L., Spitali, P., Rimessi, P., Gualandi, F., Sewry, C., et al. (2007). Dystrophin levels as low as 30% are sufficient to avoid muscular dystrophy in the human. Neuromuscul. Disord. *17*, 913–918. https://doi.org/10.1016/j.nmd.2007.07.005.
71. Sharp, P.S., Bye-a-Jee, H., and Wells, D.J. (2011). Physiological Characterization of Muscle Strength With Variable Levels of Dystrophin Restoration in mdx Mice Following Local Antisense Therapy. Mol. Ther. *19*, 165–171. https://doi.org/10.1038/mt.2010.213.
72. van Putten, M., Hulsker, M., Nadarajah, V.D., van Heiningen, S.H., van Huizen, E., van Iterson, M., Admiraal, P., Messemaker, T., den Dunnen, J.T., 't Hoen, P.A.C., and Aartsma-Rus, A. (2012). The Effects of Low Levels of Dystrophin on Mouse Muscle Function and Pathology. PLoS One *7*, e31937. https://doi.org/10.1371/journal.pone.0031937.
73. van Putten, M., Hulsker, M., Young, C., Nadarajah, V.D., Heemskerk, H., van der Weerd, L., 't Hoen, P.A.C., van Ommen, G.-J.B., and Aartsma-Rus, A.M. (2013). Low dystrophin levels increase survival and improve muscle pathology and function in dystrophin/utrophin double-knockout mice. FASEB J. *27*, 2484–2495. https://doi.org/10.1096/fj.12-224170.
74. de Feraudy, Y., Ben Yaou, R., Wahbi, K., Stalens, C., Stantzou, A., Laugel, V., Desguerre, I., FILNEMUS Network; Servais, L., Leturcq, F., and Amthor, H. (2021). Very Low Residual Dystrophin Quantity Is Associated with Milder Dystrophinopathy. Ann. Neurol. *89*, 280–292. https://doi.org/10.1002/ana.25951.
75. Godfrey, C., Muses, S., McClorey, G., Wells, K.E., Coursindel, T., Terry, R.L., Betts, C., Hammond, S., O'Donovan, L., Hildyard, J., et al. (2015). How much dystrophin is enough: the physiological consequences of different levels of dystrophin in the mdx mouse. Hum. Mol. Genet. *24*, 4225–4237. https://doi.org/10.1093/hmg/ddv155.
76. Ehrhardt, J., Brimah, K., Adkin, C., Partridge, T., and Morgan, J. (2007). Human muscle precursor cells give rise to functional satellite cells in vivo. Neuromuscul. Disord. *17*, 631–638. https://doi.org/10.1016/j.nmd.2007.04.009.
77. Chal, J., Al Tanoury, Z., Hestin, M., Gobert, B., Aivio, S., Hick, A., Cherrier, T., Nesmith, A.P., Parker, K.K., and Pourquié, O. (2016). Generation of human muscle fibers and satellite-like cells from human pluripotent stem cells in vitro. Nat. Protoc. *11*, 1833–1850. https://doi.org/10.1038/nprot.2016.110.
78. Harley, P., Paredes-Redondo, A., Grenci, G., Viasnoff, V., Lin, Y.-Y., and Lieberam, I. (2023). 3D Compartmentalised Human Pluripotent Stem Cell–derived Neuromuscular Co-cultures. Bio. Protoc. *13*, e4624. https://doi.org/10.21769/BioProtoc.4624.
79. Chen, Y., Lun, A.T.L., and Smyth, G.K. (2016). From reads to genes to pathways: differential expression analysis of RNA-Seq experiments using Rsubread and the edgeR quasi-likelihood pipeline. F1000Res. *5*, 1438. https://doi.org/10.12688/f1000research.8987.2.
80. Robinson, M.D., McCarthy, D.J., and Smyth, G.K. (2010). edgeR : a Bioconductor package for differential expression analysis of digital gene expression data. Bioinformatics *26*, 139–140. https://doi.org/10.1093/bioinformatics/btp616.
81. McCarthy, D.J., Chen, Y., and Smyth, G.K. (2012). Differential expression analysis of multifactor RNA-Seq experiments with respect to biological variation. Nucleic Acids Res. *40*, 4288–4297. https://doi.org/10.1093/nar/gks042.
82. Blighe K., Rana S., Lewis M. EnhancedVolcano: Publication-ready volcano plots with enhanced colouring and labeling. Bioconductor. 2023. doi: 10.18129/B9.bioc.EnhancedVolcano.
83. Gu, Z., Eils, R., and Schlesner, M. (2016). Complex heatmaps reveal patterns and correlations in multidimensional genomic data. Bioinformatics *32*, 2847–2849. https://doi.org/10.1093/bioinformatics/btw313.
84. Korotkevich, G., Sukhov, V., Budin, N., Shpak, B., Artyomov, M.N., and Sergushichev, A. (2021). Fast gene set enrichment analysis. Preprint at bioRxiv *60012*. https://doi.org/10.1101/060012.

STAR★METHODS

KEY RESOURCES TABLE

REAGENT or RESOURCE	SOURCE	IDENTIFIER
**Antibodies**		
Spectrin	Leica Biosystems	Cat#SPEC1-CE
Lamin A/C	Vector Laboratories	Cat#VP-L550; RRID: AB_2336546
Dystrophin (clone 2C6; MANDYS106)	Sigma-Aldrich	Cat#MABT827
Pax7	DSHB	Cat#PAX7; RRID: AB_528428
Laminin β1	Sigma-Aldrich	Cat#MAB1921P; RRID: AB_571039
CD31 (clone 390)	eBioscience	Cat#14-0311-81; RRID: AB_467200
SV2 (synaptic vesicle glycoprotein 2A)	DSHB	Cat#SV2; RRID: AB_2315387
Anti-mouse IgG2a 488	Invitrogen	Cat#A-21131; RRID: AB_141618
Anti-mouse IgG2b 594	Invitrogen	Cat#A-21145; RRID: AB_2535781
Anti-mouse IgG1 488	Invitrogen	Cat#A-21121; RRID: AB_2535764
Anti-rat IgG 546	Invitrogen	Cat#A-11081; RRID: AB_141738
α-Bungarotoxin Conjugate, Alexa Fluor 488	Invitrogen	Cat#B13422
Affini Pure Fab Fragment Donkey Anti Mouse IgG (H + L)	Jackson Immuno Research Laboratories	Cat#715-007-003; RRID: AB_2307338
**Chemicals, peptides, and recombinant proteins**		
Promocell Skeletal Muscle Growth Medium	Promocell	Cat#C-23060
Penicillin/Streptomycin	Gibco	Cat#15140122
MegaCell Dulbecco's Modified Eagle's Medium	Sigma-Aldrich	Cat#M3942
Fetal bovine serum (FBS)	Gibco	Cat#10270-098
β-Mercaptoethanol	Gibco	Cat#31350-010
MEM Non-Essential Amino Acids Solution (NEAA)	Gibco	Cat#11140-035
Glutamine (GlutaMAX™ Supplement)	Thermo Fisher	Cat#35050061
Recombinant FGF-2	PeproTech	Cat#100-18B
Fibrinogen	Sigma	Cat#F8630-1G
Matrigel	Fisher Scientific (Corning)	Cat#11543550
DMEM/F12 media	Invitrogen	Cat#11320-033
Thrombin	Sigma	Cat#T6634
Paraformaldehyde (PFA)	Thermo Scientific Chemicals	Cat#043368.9M
Goat serum	Sigma	Cat#G9023
Phosphate Buffered Saline (PBS)	Abcam	Cat#ab285410
Triton X-100	Bio-Rad	Cat#1610407
Anti-fade fluorescence mounting medium	Abcam	Cat#ab104135
DAPI (4′,6-Diamidino-2-Phenylindole, Dihydrochloride)	Sigma	Cat#D9542
Iso-pentane	Fisher Chemical	Cat#P/1030/08 CAS: 78-78-4
SYLGARD® 184 Silicone elastomer kit	SLS (Dow)	Cat#63416.5S
Pluronic® F-127	Sigma-Aldrich	Cat#P2443
UltraPure distilled water	Invitrogen	Cat#10977-035
Vetergesic (Buprenorphine hydrochloride)	National Veterinary Services	Cat#28745

(*Continued on next page*)

Continued

REAGENT or RESOURCE	SOURCE	IDENTIFIER
Metacam	National Veterinary Services	Cat#45743
Isoflurane (ISO-VET; Chanelle Pharma)	National Veterinary Services	Cat#719870
Isoflurane (ISOFLO; Abott Laboratories)	National Veterinary Services	Cat#115095
Deposited data		
Bulk RNA-seq of DMD-R3381X and CORR-R3381X myogenic cultures (previously published[33])	Gene Expression Omnibus	GSE159273
Bulk RNA-seq of DMD-K2957fs and CORR-K2957fs myogenic cultures (previously published[34])	Gene Expression Omnibus	GSE189053
Experimental models: Cell lines		
Human: CORR-R3381X MPCs (CRISPR-corrected DMD patient PSC line derived myogenic progenitor cells)	Laboratory of Yung-Yao Lin[33]	N/A
Human: CORR-K2957 fs MPCs (CRISPR-corrected DMD patient PSC line derived myogenic progenitor cells)	Laboratory of Yung-Yao Lin[34]	N/A
Human: hCD133+ (Skeletal muscle-derived CD133+ cells)	Medical Research Council Center for Neuromuscular Diseases Biobank	ID: 8206
Experimental models: Organisms/strains		
Mouse: mdx nude ($mdx^{nu}/^{nu}$)	Laboratory of Jennifer E. Morgan[8,62]	N/A
Software and algorithms		
HALO v3.6.4134 software	Indica Labs, Inc	N/A
GraphPad Prism v10	GraphPad software	N/A
STAR v2,7.0f	Dobin et al.[35]	https://github.com/alexdobin/STAR
R environment v4.2.3	R Core Team	https://cran.r-project.org
edgeR package v3.40.2	Chen et al.[77] McCarthy et al.[78] Robinson et al.[79]	https://bioconductor.org/packages/release/bioc/html/edgeR.html
EnhancedVolcano v1.16.0	Blighe et al.[80]	https://github.com/kevinblighe/EnhancedVolcano
ComplexHeatmap v2.14.0	Gu et al.[81]	https://bioconductor.org/packages/release/bioc/html/ComplexHeatmap.html
gProfiler	Kolberg et al.[36]	https://biit.cs.ut.ee/gprofiler/gost
fgsea package v1.24.0	Korotkevich et al.[82]	https://bioconductor.org/packages/release/bioc/html/fgsea.html
Other		
Biopsy punch (5mm)	Stiefel	Cat#D5245
Scalpel (size 11)	Swann-Morton	Cat#0503
Vicryl Rapide Sutures	Ethicon	Cat#W9913
Gum tragacanth	Sigma	Cat#G-112
Cryostat	Leica	Cat#CM1850 UV
Cell DIVE Multiplex Slide Scanner	Leica Microsystems	N/A
Multistainer	Leica	Cat#ST5020
Coverslipper	Leica	Cat#CV5030
Cork disks	Fisher Scientific	Cat#50-316-04
Coverslips	VWR International	Cat#631-0138
Slides	VWR	Cat#631-0108
Corning® Costar® TC-Treated Multiple Well Plates	Merck	Cat#CLS3527

(*Continued on next page*)

Continued		
REAGENT or RESOURCE	SOURCE	IDENTIFIER
6-Well CytoOne® Plate, TC-Treated	Starlab	Cat#CC7682-7506
Falcon™ Tissue Culture Dish with Grid	Fisher Scientific	Cat#10314601
VELCRO® Brand VEL-EC60217 Stick On Tape	Rapid (Velcro Europe S.A.)	Cat#54-4305
Laser cut dog-bones shaped plastic molds	Laboratory of Penney Gilbert[24]	N/A

EXPERIMENTAL MODEL AND STUDY PARTICIPANT DETAILS

Animal model

All animal experiments were conducted at the Biological Services Unit, University College London Great Ormond Street Institute of Child Health, in accordance with the Animals Act 1986, approved by the University College London Animal Welfare Ethical Review Body. Experiments were performed under Home Office licence number: PP2611161. A well-established immunodeficient *mdx* nude (*Mdx*$^{nu/nu}$) mouse model,[8,62] which harbors a nonsense mutation at the exon 23 of the dystrophin gene, lacking T cells and partial B cell deficiency, was used as host. Male and female host mice aged 5 to 8 weeks (weight 21.2–36.5 g) were used for this study. Mice of a similar age were randomly assigned to each experimental group. Immunodeficient mice were kept in individually ventilated cages in barrier conditions, in isolators or barrier containment. The colony was maintained by crossing nude females with hairy males. The nude offspring was identified by their lack of hair.

Human myogenic cells

Under appropriate ethical approvals by Hammersmith and Queen Charlotte's and Chelsea Hospital (REC ref. 06/Q0406/33) and by National Research Ethics Service Committee London-Stanmore (REC ref. 13/LO/1826; IRAS project ID: 141100), informed consent was obtained prior to the use of human derived cells.

hCD133+ cells

Healthy human skeletal muscle-derived CD133+ cells (hCD133+) were described[11] and obtained from Medical Research Council Center for Neuromuscular Diseases Biobank (ID: 8206). hCD133+ cells were maintained in Megacell Skeletal Muscle Growth Medium (For details, see Table S5).

Human PSC-derived MPCs

CORR-R3381X and CORR-K2957fs human MPCs were generated using a transgene-free myogenic differentiation protocol[77] from two precisely CRISPR-corrected DMD patient-derived PSC lines, respectively.[33,34,78] CORR-R3381X and CORR-K2957 fs MPCs were maintained in Promocell Skeletal Muscle Growth Medium (Promocell, C-23060).

METHOD DETAILS

Transcriptome analysis

Raw paired-end RNAseq data (GSE159273 and GSE189053) were aligned to the human genome (version GRCh38.104) using STAR (v2,7.0f),[35] and gene level counts quantified using the "—quantMode GeneCounts" option available with STAR. Resulting counts files were imported into an R environment (v4.2.3), along with sample meta data. Data was then processed and differential analysis performed using the edgeR package (v3.40.2).[79–81] In brief, lowly expressed genes were filtered, and samples normalised for read depth. A model matrix was created taking into account the sample genotype and group (DMD or CORR), dispersion was estimated and a generalised linear model fitted to calculate differentially expressed genes between DMD and CORR. Genes were considered differentially expressed if they had a false discovery rate (FDR) of <0.05. Volcano plots of DE genes were created using EnhancedVolcano (v1.16.0)[82] and heatmaps created using ComplexHeatmap (v2.14.0).[83] Pathway analysis using gProfiler[36] was performed by using only significant DE genes, whilst GSEA was performed using the fgsea package (v1.24.0)[84] and all genes were used after being ranked by their fold change regardless of their statistical significance, as recommended.

Manufacturing PDMS molds

24-well plates format PDMS molds were prepared as described.[24] Briefly, after weighting in and mixing proper amount of PDMS polymer and curing agent (10:1 polymer to curing agent) from the SYLGARD kit (SYLGARD 184 Silicone elastomer kit), it was put into the vacuum dessicator for 3-4 min to de-air. This process was repeated at least three times until getting rid of all the bubbles. Afterward standard 24-well cell culture plates (Merck, CLS3527) were coated with 250 μL of PDMS mixture and were put to the oven (60°C–65°C) for 30–45 min until PDMS was solidified. After curing, another 375 μL of PDMS per well was added and laser cut, dog-bone shaped acrylic molds (produced and shared by the group of Professor Penney Gilbert) were submerged in the liquid PDMS mixture. Plates were then de aired in the vacuum dessicator three times for at least 3 min. After removing bubbles, liquid PDMS

was cured at 60°C–65°C for another 30–45 min. The next step was to remove molds gently and glue three nylon hooks of Velcro fabric (Rapid, 54–4305) on both sides of the dog-bone shaped side slots of the mold using liquid PDMS mix. The 24 well plates were placed in oven again for 30–45 min for the Velcro hooks and PDMS to get solidified together. After ensuring that the Velcro hooks are glued properly to the sides of bone-shaped molds, the plates were stored with closed lid at room temperature (RT). One day before the use, PDMS plates were sterilized with 70% ethanol at RT in a tissue culture hood for at least 30 min, after which the wells were washed with sterile UltraPure distilled water (Invitrogen, 10977-035) two or three times. Afterward, 5% pluronic acid (Sigma Aldrich, P2443) solution in H_2O was added overnight and incubated at 4°C. Prior to use, pluronic acid solution was aspirated.

Cell encapsulation in hydrogel within PDMS molds

Upon cell encapsulation, CORR-R3381X and CORR-K2957fs human MPCs were expanded in Promocell medium, whereas hCD133+ cells were expanded in Megacell medium. Fibrin/Matrigel hydrogel was prepared according to the recipe (Table S5). 3D cell-laden constructs were prepared by encapsulating 10 x 10^6 cells per mL hydrogel mixture, followed by adding thrombin (Sigma, T6634; 0.2 unit per mg of fibrinogen) prior to evenly seeding cell/hydrogel suspension in PDMS molds, resulting in 0.5 x 10^6 cells/construct. 3D cell-laden constructs were then cultured in skeletal muscle growth medium. 5 days after cell encapsulation, 3D cell-laden constructs were cut with biopsy punch (Stiefel, D5245) to 5 mm in length prior to transplantation into *mdx* nude mice.

In vivo transplantation procedure

Surgery was performed under sterile conditions. Immunocompromised mdx nude mice (between 5 and 8 weeks old) were anesthetized with isoflurane (Chanelle Pharma and/or Abott Laboratories) and injected subcutaneously with Vetergesic (final dose 0.05 mg/kg) and Metacam (final dose 5 mg/kg) analgesic. The mice were kept warm during surgery that involved a longitudinal skin incision to expose the TA muscle. Afterward, a disposable scalpel size 11 (Swann-Morton, 0503) was used to cut the TA muscle longitudinally and a 5 -mm long 3D cell-laden construct was placed inside the incision. The incision was closed carefully, making sure the construct remained within the TA muscle, and the skin was sutured with Vicryl Rapide sutures (polyglactin 910, 6-0 coated, P-1, Vicryl Rapide, Ethicon W9913). All mice recovered within half an hour post transplantation without any post-operative complications.

TA muscle tissue processing

Mouse TA muscles were dissected at 4 weeks, 5 months or 6 months post transplantation, and embedded in 6% gum tragacanth (Sigma; G-112) on cork disks (Fisher Scientific, 50-316-04), before being frozen in pre-chilled isopentane in liquid nitrogen. Serial 10 μm transverse cryosections were cut throughout the muscle using a Leica CM1850 UV cryostat (Leica), collected on polylysine coated slides (VWR; 631-0108) and stored in −70°C, until analysis.

Immunocytochemistry

After taking out from −70°C and drying for 15 min at RT, slides were washed in 1X Phosphate Buffered Saline (PBS). The only time slides were fixed with 4% Paraformaldehyde (PFA) (prepared in PBS solution from 16% PFA [Thermo Scientific Chemicals; 043368.9M]) for 10 min at RT were for the Pax7/hLamininβ1/hLaminA/C/hSpectrin staining. Subsequently, slides were incubated for 1h at RT in blocking buffer containing 10% goat serum (Sigma, G9023) with 0.03% Triton X-100 (Bio-Rad, 1610407) in 1X PBS and 1:50 Affini Pure Fab Fragment Donkey Anti-Mouse IgG (Jackson Immuno Research Laboratories, 715-007-003; RRID: AB_2307338). Afterward, slides were washed 5 min in 1X PBS for three times and incubated with primary antibodies: spectrin (1:50), lamin A/C (1:500), dystrophin (1:500), laminin β1 (1:500), Pax7 (1:75), CD31 (1:100), SV2 (1:200) in 10% goat serum with 0.03% Triton X-100 in PBS overnight at 4°C. The next day, slides were washed for 5 min in PBS for three times and incubated with appropriate Invitrogen secondary antibodies: Anti-mouse IgG2a 488 (1:1000), Anti-mouse IgG2b 594 (1:1000), Anti-mouse IgG1 488 (1:1000), Anti-rat IgG 546 (1:500) in 10% goat serum with 0.03% Triton X-100 in PBS for 1h at RT. Some sections were incubated with α-Bungarotoxin conjugate (1:500). Following washing for 5 min in PBS for three times, slides were stained with DAPI (1:1000; Sigma, D9542; 10 mg/mL stock solution in H_2O) in PBS for 10 min at RT, washed another three times for 5 min in PBS and mounted with anti-fade fluorescence mounting medium (Abcam, ab104135) and covered with a coverslip. They were kept at 4°C prior to image acquisition using Cell DIVE Multiplex Slide Scanner (Leica Microsystems).

Histological staining

The Hematoxylin and eosin (H&E) staining was done using an automatic staining machine Leica Multistainer ST5020 with Coverslipper CV5030.

QUANTIFICATION AND STATISTICAL ANALYSIS

For all transplant experiments, transverse sections with the most cells and myofibers of donor origin were identified and quantified. The measurements of myofiber cross section areas (CSAs) were carried out using the annotation tool of HALO (Indica Labs, Inc) v3.6.4134. For Comparisons of CSAs measurements GraphPad Prism v10 (GraphPad software) was used to perform one-way ANOVA and Tukey's multiple comparisons test (*$p < 0.05$; **$p < 0.01$; ****$p < 0.0001$). Values were indicated as median [25th – 75th percentile]. For comparison of CSAs of donor-derived hMyofibers in mouse TA muscles from three experimental conditions, CORR-R3381 × 4 weeks (391 CSAs from 3 samples), hCD133 + 4 weeks (518 CSAs from 3 samples), and CORR-R3381X 5 & 6 months (185 CSAs from 3 samples) were compared.

Cell Reports Medicine, Volume *6*

Supplemental information

Engineered human myogenic cells in hydrogels generate innervated vascularized myofibers within dystrophic mouse muscle on long-term engraftment

Anna Kowala, James Boot, Jinhong Meng, Charles A. Mein, Olivier Pourquié, John T. Connelly, Jennifer E. Morgan, and Yung-Yao Lin

Table S1. Quantification of transplantation experiments at 4 weeks (Related to Figure 3 and 4).

Experiment no.	Timepoint of TA collection	Condition	Number of cells/myofibers detected by human-specific antibodies					
			Undifferentiated cells	Differentiated human myofibers				
			hLaminA/C+	hDys+	hSpectrin+, hDys+, hLaminA/C+	hSpectrin+, hDys+	hSpectrin+, hLaminA/C+	Total
1	4 weeks	CORR-R3381X MPCs in Promocell	2	0	2	2	0	4
			4	0	5	0	0	5
			1	0	2	0	0	2
			1	0	0	1	0	1
			10	1	16	10	0	27
			1	0	2	0	0	2
		hCD133+ in Megacell	6	1	26	17	0	44
			18	9	45	49	0	103
			9	0	1	1	0	2
			36	47	156	74	0	277
			4	11	22	20	0	53
			16	9	85	28	0	122
2	4 weeks	CORR-R3381X MPCs in Promocell	4	2	5	13	0	20
			13	13	64	100	0	177
			1	0	3	3	0	6
			5	4	6	25	0	35
			0	0	5	8	0	13
		CORR-R3381X MPCs in Megacell	0	0	0	0	0	0
			1	3	0	2	0	5
			1	0	0	2	0	2
			2	1	2	4	0	7
			0	2	2	3	0	7
			0	1	2	4	0	7
3	4 weeks	CORR-R3381X MPCs in Promocell	7	1	5	13	1	20
			10	1	16	22	0	39
			12	2	29	66	2	99
			4	3	23	88	1	115
		CORR-K295fs MPCs in Promocell	6	1	5	19	0	25
			0	0	20	40	0	60
			3	0	3	7	0	10
			2	0	3	10	0	13
			2	0	5	19	0	24
4	4 weeks	CORR-K295fs MPCs in Promocell	0	0	0	2	0	2
			12	0	0	3	0	3
			5	1	0	7	0	8
			3	0	0	2	0	2
		CORR-K295fs MPCs in Megacell	5	0	1	1	0	2
			1	2	3	5	0	10
			5	0	1	0	0	1
5	4 weeks	CORR-K295fs MPCs in Promocell	1	0	0	0	0	0
			4	0	0	0	0	0
			3	0	1	0	0	1

Table S2. Quantification of transplantation experiments at 5 and 6 months (Related to Figure 5).

Timepoint of TA collection	Condition	Number of cells/myofibers detected by human-specific antibodies						
		Undifferentiated cells		Differentiated human myofibers				
		hLaminA/C+		hDys+	hSpectrin+, hDys+, hLaminA/C+	hSpectrin+, hDys+	hSpectrin+, hLaminA/C+	Total
5 months	CORR-R3381X MPCs in Promocell	1		25	12	61	0	98
6 months	CORR-R3381X MPCs in Promocell	0		0	0	0	0	0
		0		14	6	8	0	28
		0		0	0	0	0	0
		0		22	15	22	0	59
		0		0	0	0	0	0

Table S3. Descriptive statistics for cross-section areas (CSAs) of donor-derived hMyofibers (Related to Figure 6).

	**CORR-R3381X (4 weeks)**	**hCD133+ (4 weeks)**	**CORR-R3381X (5 & 6 months)**
Total number of hMyofibers	391	518	185
CSA (μm^2)			
Minimum	26.19	21.23	29.21
25% Percentile	181.6	101	174.5
Median	317.2	172.5	344.5
75% Percentile	494.7	302.6	577.5
Maximum	2933	1600	3055
Range	2906	1579	3025
Mean	400.3	242.7	420.8
Std. Deviation	334.4	223.7	351.4
Std. Error of Mean	16.91	9.828	25.84

Table S4. Relative frequency distribution (percentage) (Related to Figure 6).

Bin Center (µm^2)	CORR-R3381X (4 weeks) (%)	hCD133+ (4 weeks) (%)	CORR-R3381X (5 & 6 months) (%)
50	15.08951407	44.59459459	17.83783784
250	40.15345269	35.52123552	32.97297297
450	26.342711	12.93436293	21.62162162
650	7.16112532	3.474903475	14.59459459
850	5.626598465	1.544401544	5.945945946
1050	2.046035806	0.772200772	4.324324324
1250	1.023017903	0.386100386	1.621621622
1450	1.023017903	0.386100386	0
1650	0.767263427	0.386100386	0.540540541
1850	0	0	0
2050	0.511508951	0	0
2250	0	0	0
2450	0	0	0
2650	0	0	0
2850	0.255754476	0	0
3050	0	0	0.540540541

Table S5. Cell culture medium and hydrogel recipes (Related to Figure 2 - 6).

#	Name	Details
1	Promocell Skeletal Muscle Growth medium (C-23060)	95 ml of basal medium (C-23060B), 5 ml of Supplement Mix (C-39365) and 105 µl 0.2% Penicillin/Streptomycin (Gibco, 15140122)
2	Megacell Skeletal Muscle Growth Medium	MegaCell Dulbecco′s Modified Eagle′s Medium (Sigma-Aldrich, M3942) supplemented with 10% fetal bovine serum (FBS, Gibco, 10270-098), 0.1mM β-Mercaptoethanol (Gibco, 31350-010), 1% NEAA (Gibco, 11140-035), 2µM Glutamine (GlutaMAX™ Supplement, Thermo Fisher, 35050061) and 5 ng/ml recombinant FGF-2 (PeproTech, 100-18B)
3	Fibrin/Matrigel hydrogel	4 mg/ml fibrinogen (40% vol; Sigma, F8630-1G), Matrigel (3.5 mg/ml; Fisher Scientific, 11543550) in DMEM/F12 media (40% vol; Invitrogen, 11320-033), thrombin (Sigma, T6634; 0.2 unit/mg fibrinogen)
